# The Penn State Protein Ladder system for inexpensive protein molecular weight markers

**DOI:** 10.1038/s41598-021-96051-x

**Published:** 2021-08-18

**Authors:** Ryan T. Santilli, John E. Williamson, Yoshitaka Shibata, Rosalie P. Sowers, Andrew N. Fleischman, Song Tan

**Affiliations:** 1 Center for Eukaryotic Gene Regulation, Department of Biochemistry and Molecular Biology, The Pennsylvania State University, University Park, PA 16802 USA; 2 Schreyer Honors College, The Pennsylvania State University, University Park, PA 16802 USA; 3 Present Address: Thomas Jefferson University, 111 South 11th Street, Suite 8490 Gibbon, Philadelphia, PA 19107 USA

**Keywords:** Biochemistry, Genetic engineering, Immunoblotting, Electrophoresis, Protein purification

## Abstract

We have created the Penn State Protein Ladder system to produce protein molecular weight markers easily and inexpensively (less than a penny a lane). The system includes plasmids which express 10, 15, 20, 30, 40, 50, 60, 80 and 100 kD proteins in *E. coli*. Each protein migrates appropriately on SDS-PAGE gels, is expressed at very high levels (10–50 mg per liter of culture), is easy to purify via histidine tags and can be detected directly on Western blots via engineered immunoglobulin binding domains. We have also constructed plasmids to express 150 and 250 kD proteins. For more efficient production, we have created two polycistronic expression vectors which coexpress the 10, 30, 50, 100 kD proteins or the 20, 40, 60, 80 kD proteins. 50 ml of culture is sufficient to produce 20,000 lanes of individual ladder protein or 3750 lanes of each set of coexpressed ladder proteins. These Penn State Protein Ladder expression plasmids also constitute useful reagents for teaching laboratories to demonstrate recombinant expression in *E. coli* and affinity protein purification, and to research laboratories desiring positive controls for recombinant protein expression and purification.

## Introduction

Protein ladders or molecular weight markers are among the most commonly used reagents in biochemistry experiments. They provide molecular weight standards to estimate the size of proteins separated by gel electrophoresis like SDS-PAGE (sodium dodecyl sulfate polyacrylamide gel electrophoresis). As such, protein ladders constitute critical reference reagents when expressing, purifying or analyzing proteins.

Early protein ladders were comprised of readily available proteins such as lysozyme (14 kD), soybean trypsin inhibitor (21 kD), carbonic anhydrase (31 kD), ovalbumin (45 kD), serum albumin (67 kD) and phosphorylase b (97 kD). These native protein ladders were commercially available and relatively inexpensive at about US$ 0.10 per lane. Such ladders have been replaced more recently by recombinant ladders with rounded molecular weights (e.g. 25, 50 kD) and with optional features such as prestaining with dyes for visibility during electrophoresis and on Western blots. These improvements have come at an increased expense with most commercially available unstained ladders costing about US$ 1.00 per lane.

There are a few reported examples of home-made protein ladders. Doucet and Beauregard produced a protein ladder by disulfide crosslinking a 11 kD designer protein via oxidation in solution^1^. Other home-made protein ladders have tackled the problem of detecting protein ladders on Western blots. The traditional method to visualize protein ladders on Western blots is to use prestained protein ladders which remain visible when transferred to the blotted membrane. The Western blot X-ray film (if using chemiluminescence) can be overlaid onto the blotted membrane and the positions of the protein ladder bands marked by pen on the X-ray film. To avoid this inelegant approach, protein ladders have been created to contain peroxidase activity (for detection by peroxidase-mediated chemiluminescence)^2^, a medley of 14 affinity tags (to bind anti-tag antibodies)^3^, or fragments of or intact IgG binding domains (for detection by peroxide-linked second antibodies)^4^. An alternate approach allows prestained markers to be detected in Western blots using monoclonal antibodies generated against the Remazol dyes present in prestained proteins (these monoclonal antibodies are now commercially available from Diagenode)^5^.

We have previously described two plasmids that can be used to prepare both 100 bp and 1000 bp DNA molecular weight markers efficiently and inexpensively^6^. We believe there is a similar need for inexpensive protein molecular weight markers, and we have therefore created bacterial expression vectors to express ladder proteins that range from 10 to 100 kD. These proteins can be expressed at very high levels in *E. coli* and purified by metal affinity chromatography with minimal equipment. The ladder proteins can be detected by Coomassie Blue staining of SDS-PAGE gels and on Western blots through engineered IgG binding domains. We have also constructed polycistronic expression vectors to coexpress either the set of 10, 30, 50, 100 kD proteins or the set of 20, 40, 60, 80 kD proteins, enabling the efficient production of a 10–100 kD protein ladder with just two expression experiments.

## Results

### Design considerations

We designed our protein ladder to be comprehensive as well as simple and inexpensive to prepare. We identified the following criteria for the ladder proteins:

1. The basic ladder should be comprised of 10, 20, 30, 40, 60, 80 and 100 kD proteins, with additional 15, 150 and 250 kD proteins.
2. The individual proteins should migrate as close as possible to the appropriate position on SDS-PAGE gels.
3. The individual proteins should express solubly in *E. coli* at very high levels, i.e. > 10 mg/L of culture.
4. The individual proteins should be capable of being expressed at temperatures around 20–25 °C to obviate the need for temperature-controlled incubators.
5. The individual proteins should be purifiable with high purity and high yields using metal affinity chromatography resin.
6. The individual proteins should be purifiable at sufficiently high concentrations so that protein ladders can be created by simply mixing the metal affinity eluents, i.e. no need to concentrate the metal affinity eluents.
7. The individual proteins should be tagged with IgG binding domains to allow detection on Western blots without the need for specialized antibodies.
8. The proteins in the basic ladder should be capable of being coexpressed for efficient production of 10–100 kD ladders.

We achieved criterion 1 by combining protein motifs and domains, adding short affinity tags or linker sequences as necessary to produce the desired molecular weight. Criterion 2 is an obvious requirement but was not trivial to achieve. Despite our attempt to use proteins for which we could find published evidence for appropriate mobility on SDS-PAGE, many proteins, particularly lower molecular weight ones, migrated anomalously. Criterion 3 that the proteins be expressed in *E. coli* at very high levels was critical for at least two reasons. Firstly, very high level expression increases the efficiency and decreases the cost of producing the protein ladder. Secondly, very high expression is functionally equivalent to additional purification steps, rendering a single purification step by metal affinity chromatography sufficient. To achieve criteria 2 and 3, we combined our previous experience expressing and purifying proteins in *E. coli* with literature and internet searches, and soliciting ideas from the CCP4 crystallographic mailing list. For criterion 7, we had previously confirmed that the IgG binding domains of *S. aureus* protein A^7^ could be detected in Western blotting using a peroxidase-linked second antibody^8^. For the coexpression requirement of criterion 8, we repurposed our modular pST44 polycistronic expression system originally designed to reconstitute protein complexes by coexpression in vivo^9^.

### Expression of individual ladder proteins

Our efforts to express the individual proteins progressed in three phases. We first expressed what we thought should be appropriate recombinant protein constructs with the desired molecular weights. Unfortunately, many of the proteins between 10 and 30 kD migrated anomalously on SDS-PAGE, necessitating expression of additional protein constructs (described in Supplementary Note 1). In the final phase, we reengineered most of the ladder proteins to include tandem IgG binding domains when we observed that a single IgG binding domain was poorly detected. The final ladder proteins and corresponding expression plasmids are listed in Table 1, while the protein sequence of each ladder protein is pr

**Table 1:** Penn State ladder proteins and expression plasmids.

nominal MW (kD)	Expected MW (kD)	Identity	Expression plasmid
10	10.01	STRHSTPAB	pPSU10
15	15.01	HSTPABPAC	pPSU15
20	19.88	STRHSTPABPACCBPv3	pPSU20
30	29.78	HSTPABPACS100BCBP	pPSU30
40	40.36	HSTPABPACGST	pPSU40
50	50.31	STRHSTPABMBP	pPSU50
60	60.03	HSTPABPACRCC1	pPSU60
80	79.83	STRHSTPABPACIL1bRCC1	pPSU80
100	98.27	STRHSTPABPACIL1bQRS	pPSU100
150	149.95	STRHSTPABMBPpepN	pPSU150
250	249.90	STRHSTPABPACIL1bQRS- STRHSTPABMBPpepN	pPSU250
10 + 30 + 50 + 100			pPSU10-30–50-100
20 + 40 + 60 + 80			pPSU20-40–60-80

ovided in Supplementary Note 2.

**Figure 1:**
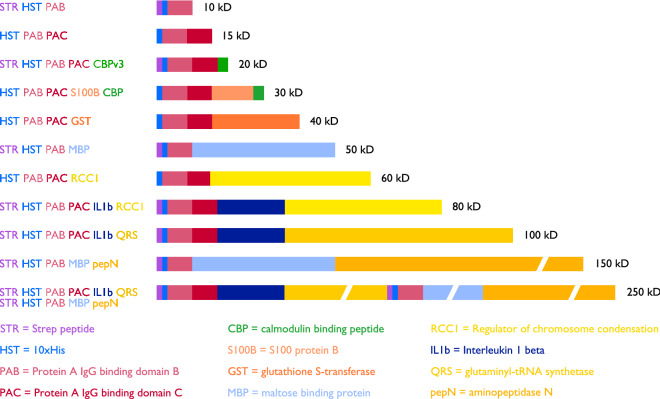
Schematic representation of Penn State ladder proteins with abbreviation key and color coding shown

Figure 1 shows the design of the 11 ladder proteins from 10 to 250 kD. Each protein contains a decahistidine tag (HST = HIS Ten) for metal affinity purification and the *S. aureus* Protein A IgG binding domain B (PAB) for Western blot detection. Our selection of the different protein segments in the ladder proteins was informed by a variety of sources. We employed commonly used proteins such as GST (glutathione S-transferase) and MBP (maltose-binding protein) because they were known to express at high levels in *E. coli* and we had observed them to migrate appropriately on SDS-PAGE. We knew that the RCC1 (regulator of chromosome condensation) protein expressed at high levels in *E. coli* and migrated appropriately on SDS-PAGE from our studies of this protein^10,11^. Our previously investigations of the Strep (Strep-tag), HPC (heavy chain of protein C) and CBP (calmodulin binding peptide) affinity tags indicated that these tags neither adversely affect expression nor seemed to cause anomalous mobility^12^. We selected glutaminyl-tRNA synthetase (QRS)^13,14^ and aminopeptidase N (pepN)^15^ for study by searching the RCSB Protein Data Bank for crystal structures of large *E. coli* proteins, reasoning that such proteins were likely to express well in *E. coli* to produce enough material for crystallization studies and crystal structure determination. The interleukin 1 beta protein (IL1b)^16^ was proposed by Dr. Erik Klontz from the University of Maryland School of Medicine after we solicited suggestions from the CCP4 crystallographic mailing list. We discovered the S100B protein^17^ when browsing an online catalog which showed the purified protein on an SDS-PAGE gel together with the calculated molecular weight.

We had originally planned to limit our ladder proteins to 100 kD or smaller, reasoning that proteins larger than 100 kD are usually difficult to express in *E. coli*. However, our finding of a report that the 99 kD aminopeptidase N enzyme could be expressed at 75 mg/L culture^15^ encouraged us to create a 150 kD ladder protein by fusing the 50 kD MBP ladder protein to aminopeptidase N. When we observed reasonable expression of this 150 kD ladder protein, we were emboldened to fuse our 100 and 150 kD ladder proteins to create a 250 kD protein.

**Figure 2:**
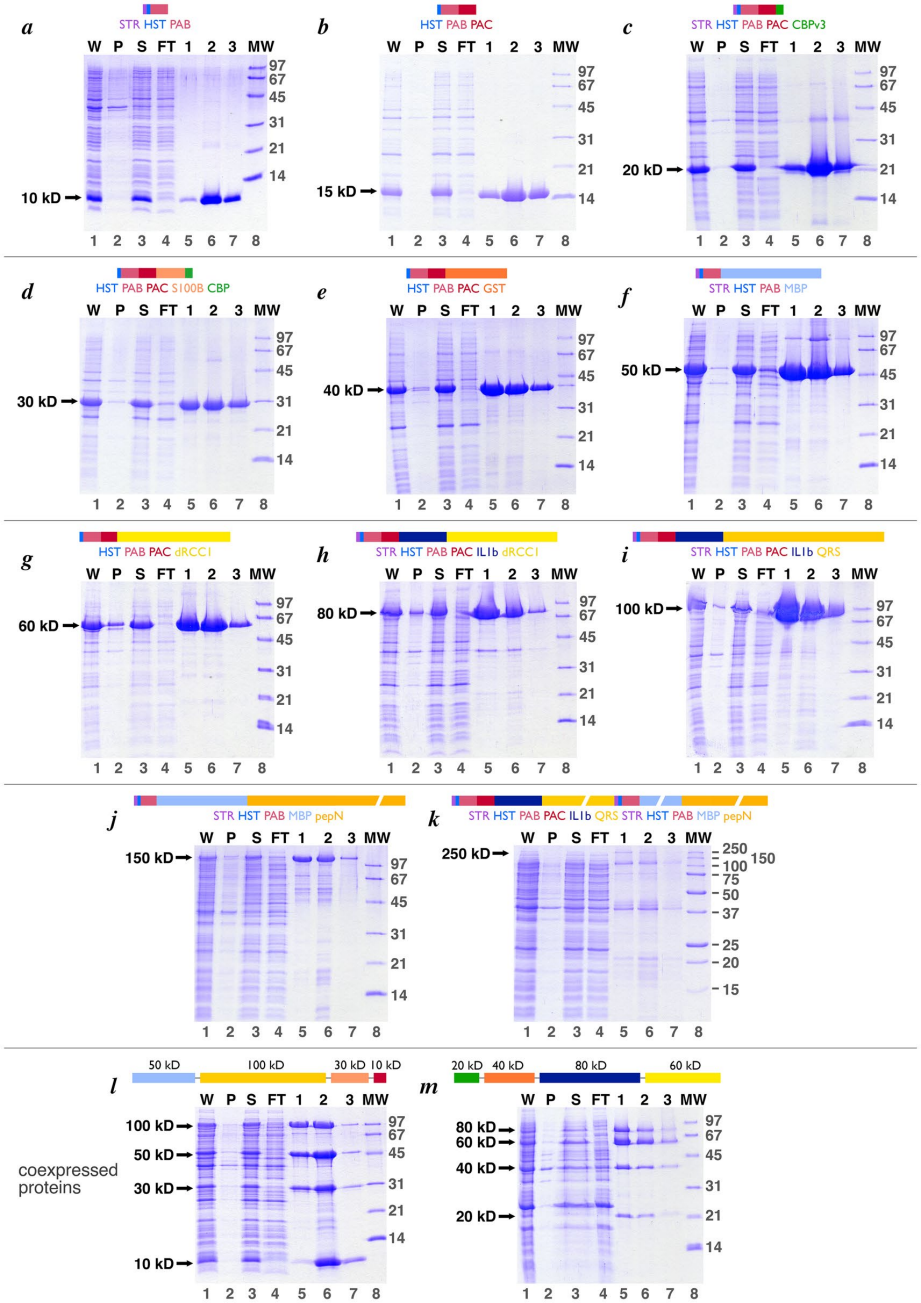
Purification of Penn State ladder proteins by metal affinity chromatography. Panels (**a**) through (**k**) show metal affinity purification of the individual 10, 15, 20, 30, 40, 50, 60, 80, 100, 150 and 250 kD ladder proteins respectively, while panels (**l**) and (**m**) show the equivalent for the coexpressed 10, 30, 50, 100 kD and the coexpressed 20, 40, 60, 80 ladder proteins respectively. The schematic for each individual protein is shown above each gel, while the schematic showing the order of expression in the coexpression constructs are shown for panels l and m. In each panel, lanes 1 through 7 show the whole cell extract (W), an equivalent volume sample of the pellet fraction after lysis (P), an equivalent volume of the supernatant fraction after lysis (S), an equivalent volume sample of the metal affinity flow through fraction (FT), and the metal affinity elution fractions 1, 2 and 3. The volumes of the whole, pellet, supernatant and flow through samples were approximately 5 ml each, while the volumes of the elution fractions were approximately 0.5 ml each. 5 µl of a 1:4 dilution of each sample was loaded onto 18% acrylamide SDS-PAGE gels and stained with Coomassie Blue. Lane 8 of all panels except for panel k show the Bio-Rad low molecular weight (LMW) markers, while lane 8 of panel k shows the Bio-Rad Precision Plus markers. The location of the band for each protein is shown with an arrow to the left of each gel.

We employed our *E. coli* expression vector, pST50Tr, because its modular design simplified the creation of expression plasmids with shared elements such as the tandem decahistidine tag-Protein A IgG binding domain B (HST-PAB). pST50Tr is a T7-promoter based plasmid which confers ampicillin resistance^9^. We expressed individual proteins in 100 ml of culture and prepared soluble extracts by sonication. The proteins were purified by incubating the soluble extract with Talon metal affinity resin in batch before transferring the resin to a disposable mini-column and eluting the protein manually with imidazole. Representative metal affinity purification of the 11 ladder proteins are shown in Fig. 2a–k. All 11 proteins are expressed solubly with only a small amount of protein in the pellet fraction of the extract (lanes 2). Incubation of the soluble extract with the metal affinity for 20 min at room temperature was sufficient for efficient capture, as evidenced by the absence of each ladder protein in the unbound (FT or flow through) fraction (compare lanes 3 and 4 for each gel). It is worth noting that 0.5 ml of Talon resin was sufficient to bind ladder protein from 50 ml of culture, even when the ladder protein was heavily overexpressed. Each of the 10, 15, 20, 30, 40, 50, 60, 80 and 100 kD ladder proteins was substantially purified by this single metal affinity step, with contaminating proteins constituting a very small fraction of the total protein. We estimate that these ladder proteins are expressed at levels of 10–50 mg/L of culture based on Coomassie stained band intensities on SDS-PAGE and larger scale expression/purifications we have performed. Not surprisingly, the 150 and 250 kD ladder proteins showed significant smaller molecular weight contaminants, presumably degradation products of the full length ladder protein. The metal affinity purified 150 kD ladder protein is usable (Fig. 2j) although the smaller molecular weight contaminants might be faintly visible in the final molecular weight ladder. In contrast, the 250 kD ladder protein is expressed much less efficiently and constitutes less than 5% of the metal affinity eluted protein (Fig. 2k). Consequently, the 250 kD protein will be less effective as a ladder protein than the others.

**Figure 3:**
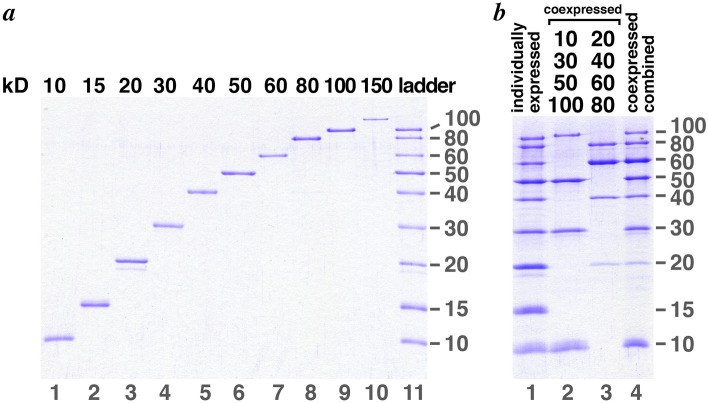
The Penn State ladder proteins, individually expressed or coexpressed. (**a**) The same metal affinity purified individual proteins presented in Fig. 2 are shown in lanes 1 through 10. Lane 11 shows the 10-100 kD ladder assembled from the same samples shown in lanes 1 through 9 at the same concentrations. Individual proteins from the most concentrated metal affinity fractions were diluted with protein gel loading buffer as shown in parentheses: 10 kD (1:80), 15 kD (1:120), 20 kD (1:160), 30 kD (1:60), 40 kD (1:120), 50 kD (1:120), 60 kD (1:80), 80 kD (1:80), 100 kD (1:120), 150 kD (1:20). (**b**) Coexpressed Penn State ladder proteins. Lane 1 shows a ladder assembled from individually expressed and purified ladder proteins similar to that in panel (a) lane 11. Lane 2 shows the metal affinity purified coexpressed 10, 30, 50 and 100 kD ladder proteins (1:30 dilution of original fraction shown as 1:4 dilution in Fig. 5a, lane 6), while lane 3 shows the equivalent for the coexpressed 20, 40, 60 and 80 ladder proteins (1:20 dilution of original fraction shown as 1:4 dilution in Fig. 5b, lane 5). Lane 4 shows the coexpressed 10-100 kD ladder assembled by mixing the coexpressed 10, 30, 50, 100 kD and the coexpressed 20, 40, 60, 80 kD metal affinity fractions.

Figure 3a shows the same individually expressed and purified proteins in Fig. 2a–k together with the corresponding protein ladder combining the 10, 15, 20, 30, 40, 50, 60, 80, 100 kD proteins. We refer to all combinations of our ladder proteins as “Penn State protein ladder”. The 10 to 100 kD samples in Fig. 3a correspond to 3 µl of 1:60 to 1:160 dilutions of the most concentrated metal affinity elution fraction, where each elution fraction had a volume of ~ 0.5 ml. For the 150 kD protein, 3 µl of a 1:20 dilution of the metal affinity fraction 1 was used. Since the metal affinity purification yielded approximately 1 ml for each protein (2 fractions of 0.5 ml), each lane in Fig. 3a corresponds to 0.002 to 0.005% of the purified protein. In other words, 50 ml of culture yields enough protein for 20,000 to 50,000 lanes of each ladder protein, and 50 ml cultures for each of the 9 proteins from 10 to 100 kD is sufficient to produce 20,000 lanes of the Penn State protein ladder shown in Fig. 3a lane 11. Even though the 150 kD protein is expressed at lower levels compared to the other smaller proteins, enough of this protein was purified from 50 ml of culture for 6,700 lanes.

**Figure 4:**
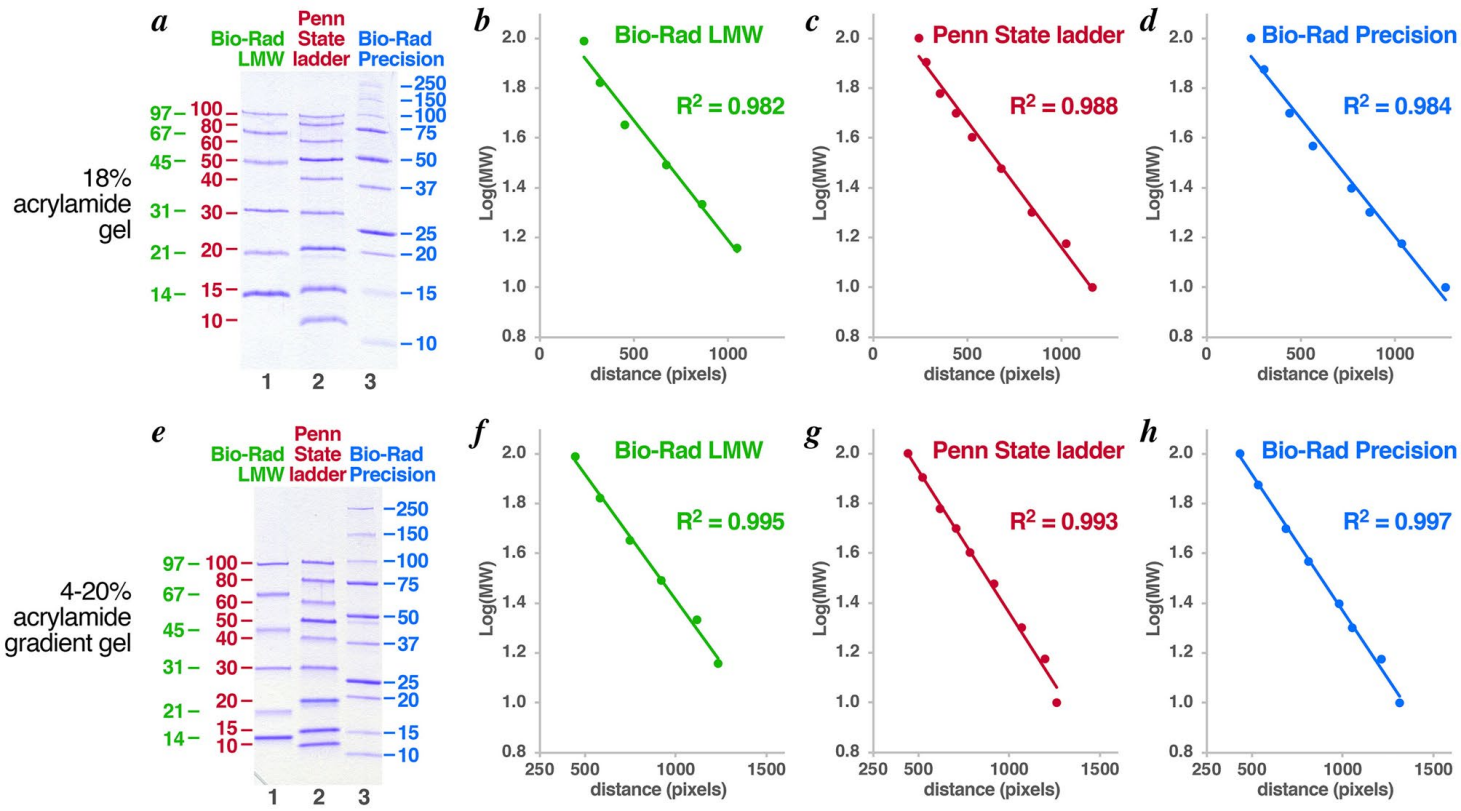
Comparison of Penn State protein ladder with commercial protein ladders. (**a**) SDS-PAGE of Bio-Rad low molecular weight (LMW) markers (lane 1, green), Penn State 10-100 kD ladder (lane 2, red) and Bio-Rad Precision Plus protein ladder (lane 3, blue) on 18% acrylamide gel. Sizes of bands in kD are shown to the sides. Semi-log plots of the LMW markers, Penn State ladder and Precision Plus ladder with R^2^ goodness of fit for the samples shown in panel (**a**) are shown in panels (**b**), (**c**) and (**d**) respectively. The equivalent panels for samples electrophoresed through a 4-20% acrylamide gradient gel (Bio-Rad, 456-1093) are shown in panels (**e**) through (**h**).

We compared the performance of the Penn State protein ladder with commercial ladders on 18% acrylamide and 4–20% acrylamide gradient SDS-PAGE gels (Fig. 4). We find that the Bio-Rad low molecular weight (LMW) markers (green), the Penn State protein ladder (red) and the Bio-Rad Precision Plus protein markers (blue) produce similar results in semi-log plots (evaluated for proteins up to 100 kD). The Penn State ladder has a slightly better goodness of fit (0.988 vs 0.982 for the Bio-Rad LMW vs 0.984 for Bio-Rad Precision Plus) on an 18% acrylamide gel. All three ladders produced excellent goodness of fit on the 4–20% acrylamide gradient gel (0.995 for the Bio-Rad LMW, 0.993 for the Penn State protein ladder and 0.997 for the Bio-Rad Precision Plus). Although specific proteins might migrate less appropriately in each of the three ladders, the overall performance of the three ladders was largely equivalent.

### Coexpression of ladder proteins

To produce protein ladders even more efficiently, we employed our pST44 polycistronic expression system to coexpress 8 ladder proteins (10, 20, 30, 40, 50, 60, 80, 100 kD) on two plasmids. This modular polycistronic expression system uses the same pST50Tr plasmids which express individual proteins as the source of translational cassettes to construct polycistronic expression vectors^9^. Since the individual ladder proteins were already cloned in pST50Tr plasmids, a series of straightforward subcloning steps produced two polycistronic expression vectors, each coexpressing ladder proteins. The first coexpresses the 10, 30, 50, and 100 kD proteins at equivalent levels (Fig. 2l). As before, all proteins were expressed solubly and were purified by metal affinity with excellent yields. The initial version of the second polycistronic plasmid to coexpress the 20, 40, 60 and 80 kD ladder proteins reproducibly produced less satisfactory results: the four proteins were not expressed or purified at equivalent levels with significantly more 60 kD proteins purified compared to the other proteins (data not shown). Furthermore, the purity of the metal affinity purified material was relatively poor. Since each of the four proteins could be expressed and purified to very high levels individually, we considered the effect of changing the order of expression in the polycistronic expression vector. Instead of expressing the proteins in order of 60, 80, 40 and 20 kD proteins, we constructed a pST44 polycistronic vector with the order changed to 20, 40, 80 and 60 kD (this order was partly constrained by restriction sites internal to the coding regions which would complicate subcloning the individual translational cassettes). This new polycistronic vector coexpressed the 20, 40, 60 and 80 kD proteins with improved purity, although the 20 and 40 kD proteins are expressed at lower levels than the 80 kD and particularly the 60 kD proteins (Fig. 2m).

The metal affinity purified fractions for the coexpressed 10, 30, 50 and 100 kD proteins and the coexpressed 20, 40, 60 and 80 kD proteins can be combined to produce a protein ladder. This coexpressed protein ladder is comparable to the individually expressed protein ladder with two differences: the coexpressed protein ladder lacks the 15 kD protein and the 20 kD protein band in the coexpressed protein ladder is relatively weak. These shortcomings may be acceptable considering the improved efficiency from two versus nine expression and purification procedures. The samples in Fig. 3b lanes 2–4 correspond to 4 µl of a 1:30 dilution (for the 10, 30, 50, 100 kD proteins) or 1:20 dilution (for the 20, 40, 60, 80 kD proteins) of the 0.5 ml fraction or about 0.04% of the most concentrated metal affinity purified fraction. Thus, this fraction is enough for at least 2500 lanes. Since approximately 50% more purified material is additionally found in the second most concentrated metal affinity fraction (fractions 1 in Fig. 2l,m), enough material can be purified for 3,750 lanes from two 50 ml cultures (one for each of the two polycistronic expression plasmids). This is a comparable yield per volume of culture to the 20,000 lanes from eight 50 ml cultures if the proteins were expressed individually.

### Ladder proteins in western blotting

**Figure 5:**
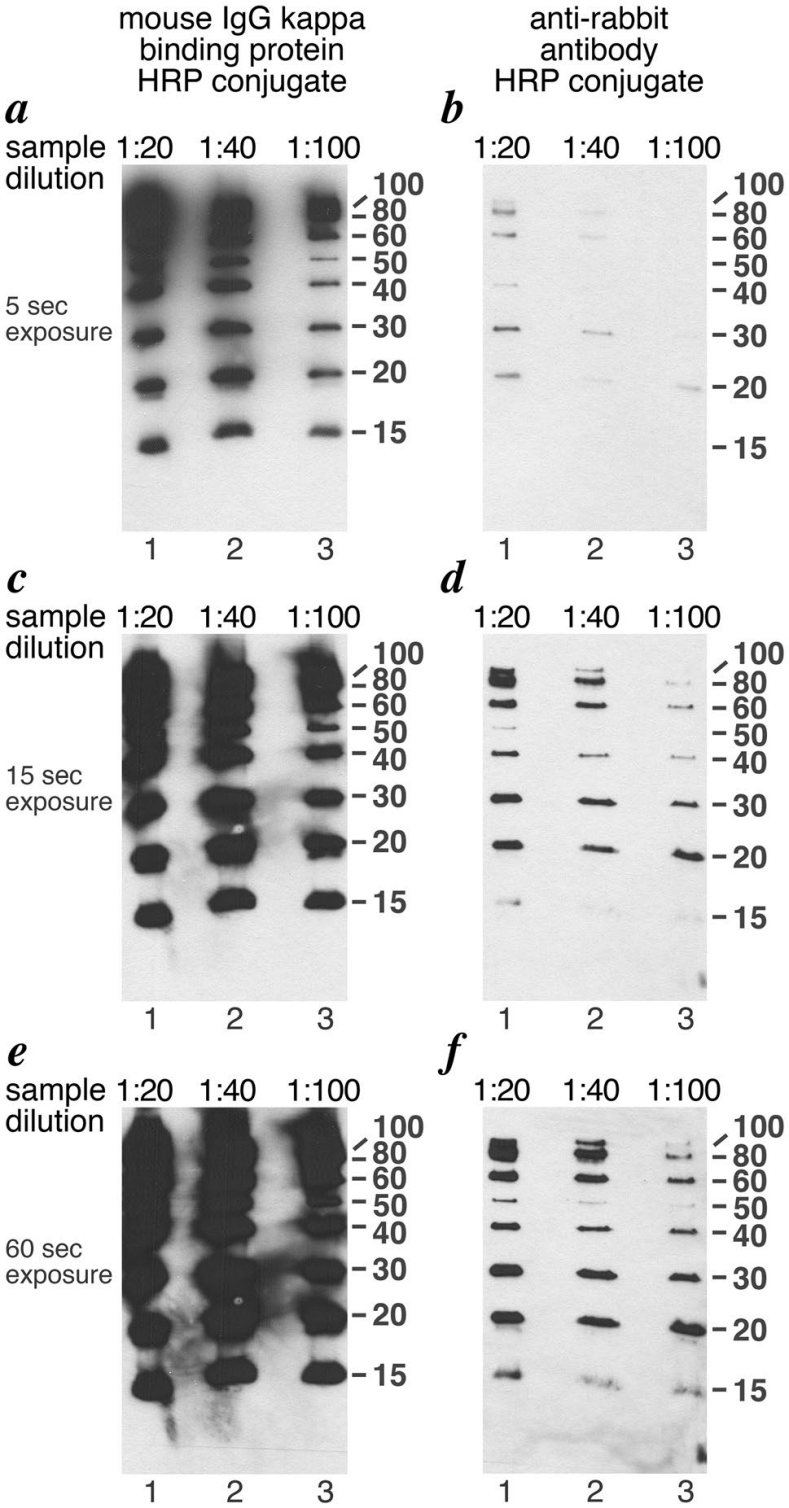
Use of Penn State protein ladder in Western blots. The Penn State ladder containing 10, 15, 20, 30, 40, 50, 60, 80 100 kD proteins are shown in each panel at (**a**) 1:20, 1:40 and 1:100 dilution in lanes 1, 2 and 3 respectively. Panels (**a**), (**c**) and (**e**) show the ladders detected via the Protein A IgG binding domain(s) using a 1:5000 dilution of the mouse IgG kappa binding protein HRP conjugate (Santa Cruz sc516102) while panels (**b**), (**d**) and (**f**) were detected using a 1:5000 dilution of the HRP-linked donkey anti-rabbit IgG (Cytiva NA934-1ML). The blots were developed using SuperSignal West Pico PLUS chemiluminescent substrate (ThermoFisher 34580) and exposed to X-ray film for 5 sec (panels **a** and **b**), 15 sec (panels **c** and **d**) or 60 sec (panels **e** and **f**).

We examined the performance of the nine protein Penn State protein ladder in Western blotting using either HRP-conjugated mouse IgG kappa binding protein (Santa Cruz Biotechnology) or HRP-conjugated anti-rabbit whole IgG (Cytiva) to detect the IgG binding domains. Each of the 15 to 100 kD ladder proteins is detected in Western blots experiments (Fig. 5). The 50 kD ladder protein is detected more weakly due to the presence of only one IgG binding domains compared to the two tandem IgG binding domains in the other 15 to 100 kD ladder proteins. We anticipated a weak signal for the 10 kD ladder protein, which also contains only one IgG binding domain, but we do not detect this protein by Western blotting. We have not investigated the reason for this, and we cannot eliminate the possibility that the 10 kD protein did not transfer well.

As expected, Western blotting is able to detect much smaller quantities of the ladder proteins than Coomassie staining. When using the HRP-conjugated mouse IgG kappa binding protein, a 1:50 dilution of the ladder proteins equivalent to what is shown in Fig. 3, lane 10 can be detected in a 5 s exposure by chemiluminescence. Use of anti-rabbit whole IgG required about 60 s exposure to detect each of the 15 to 100 kD ladder proteins at this 1:50 dilution or about 15 s exposure of a 1:10 dilution. Such longer exposures using mouse IgG kappa binding protein were overexposed and significantly higher dilutions of the ladder proteins should be used if long exposures are required. The ability to detect a 50 × dilution of the proteins suggests that 50 ml of culture for each of the individual expression plasmids will be enough for at least 50 times 20,000 or 1,000,000 lanes of ladder protein in Western blotting experiments.

## Conclusion

We have developed two simple and efficient ways to prepare 10 to 100 kD protein molecular weight markers appropriate for both Coomassie staining and Western blotting. The 10, 15, 20, 30, 40, 50, 60, 80, 100 and 150 kD proteins can be expressed individually and purified using a one-step metal affinity purification procedure. This approach allows one to select which proteins to include in the protein ladder and it also allows one to control the intensity of the individual bands in the ladder. For example, one could increase the concentrations of one or more specific proteins to act as fiducial marks. We use 4 × of the 50 kD ladder protein within our laboratory for this purpose because this protein is roughly in the middle of the 10 to 100 kD range and because it is among the mostly highly expressed of the proteins. Expressing and purifying individual proteins also permits specific proteins to be prestained with dyes using inexpensive textile dyes^18^. Our alternate approach for ladder production uses the polycistronic expression plasmids for the 10, 30, 50, 100 and 20, 40, 60, 80 proteins to prepare a protein ladder from just two expression cultures. This coexpression approach is more efficient for preparing an adequate protein ladder, although with a weaker 20 kD band in the resulting ladder.

The protein ladders can be expressed and purified with minimal laboratory equipment. We selected proteins that can be expressed at ambient temperatures (21 to 25 °C) to reduce the need for a controlled temperature incubator. We used sonication to lyse bacterial cells to release proteins and to fragment chromosomal DNA, but the combination of detergents and nucleases should work as well. A low speed centrifuge to pellet the cells from the growth culture and a microcentrifuge to clear the lysate are the only other equipment needed.

A major benefit of the Penn State protein ladder system is the inexpensive cost (Table 2). We estimate it costs about US$ 8 for us to express and purify proteins from 50 ml of culture, with the major costs being the metal affinity resin (~ US$ 6) and the disposable spin column (~ US$ 2). For US$ 72 (9 × US$ 8), one can purify enough of the 9 individual ladder proteins (10, 15, 20, 30, 40, 50, 60, 80, 100 kD) for 20,000 lanes, or $0.0036 per lane. Similarly, one can purify enough of the 8 coexpressed proteins (10, 20, 30, 40, 50, 60, 80, 100) for 3,750 lanes for US$ 16 or $0.0043 per lane. In comparison, commercial ladders average about US$ 1.00 per lane, although it should be noted that many commercial ladders do include 150 and 200 or 250 kD bands. Thus, the Penn State protein ladders can be prepared for less than a penny a lane or 1/100th the cost of commercial protein ladders. The cost is even less if one recycles the metal affinity resin as we are a

**Table 2:** Comparison of reagent costs to prepare protein ladders.

	cost	# lanes	cost/lane	cost/100 lanes
Penn State 9 individual proteins	$ 72	20,000	$ 0.0036	$ 0.36
Penn State 8 coexpressed proteins	$ 16	3,750	$ 0.0043	$ 0.43
Bio-Rad Precision Plus	$ 105	100	$ 1.05	$ 105
NEB unstained protein standard	$ 133	150	$ 0.89	$ 89
Thermo Scientific PageRuler	$ 108	100	$ 1.08	$ 108

ble to do in our laboratory.

We anticipate that the expression plasmids for the Penn State protein ladder system will have uses beyond the primary use for protein molecular weight markers. The ability to prepare proteins of well-defined molecular weights may be useful as protein standards for disciplines such as mass spectrometry. Being able to express large quantities of proteins in *E. coli* may be helpful to research laboratories that do not perform *E. coli* overexpression or purification of recombinant proteins on a regular basis and that would like positive controls to help troubleshoot such expression experiments. High school and college teaching laboratories may also find the robust expression of the proteins and the minimal laboratory equipment needed for purification appropriate for incorporating expression and purification experiments into their curriculum.

We would like the scientific community to make use of the Penn State protein ladders, and we are therefore making available the protein ladder expression plasmids to the nonprofit academic community without licensing requirements. The expression plasmids for the Penn State protein ladders are being deposited in the Addgene and DNASU plasmid repositories to facilitate distribution. Detailed instructions for preparing the Penn State protein ladders are provided in Supplementary Note 4.

## Methods

### Plasmid constructs

Standard recombinant DNA procedures were used to construct *E. coli* expression vectors for the ladder proteins. The following coding cassettes were synthesized as IDT gBlock double-stranded DNA fragments: STRHSTPAB, HSTPABPAC, S100B, IL1b, CJ and hTrx2. Drosophila RCC1 residues 4–422 were amplified from pWM529-dRCC1(10–422) × 27, a derivative of the pST50Tr-STRaHISNdRCC1t1 plasmid used to express Drosophila RCC1 for crystallization studies^11,19^. The glutaminyl-tRNA synthetase (QRS) and aminopeptidase N (pepN) coding regions were amplified from *E. coli* genomic DNA using Q5 DNA polymerase (Biolabs). Expression plasmids were constructed using the modular pST50Tr T7 promoter-based expression plasmid^9^. Individual regions were cloned in the 5’ Nde-BamHI region, the middle BamHI-BsrGI region or the 3’ BsrGI-NgoMIV region. For constructs requiring more than three regions, restrictions sites with sticky ends compatible with BamHI (e.g. BglII) or NgoMIV (e.g. AgeI or BspEI) were used to destroy these original restriction sites together with primers which recreated these restriction sites elsewhere. The expression plasmids for the individual ladder proteins range from 3082 bp (pPSU10 to express the 10 kD protein**)** to 9457 bp (pPSU250 to express the 250 kD protein). The sizes of the polycistronic expression vectors are 8063 bp (pPSU10-30-50-100 to coexpress the 10, 30, 50, and 100 kD ladder protein) and 8438 bp (pPSU20-40-60-80) to express the 20, 40, 60, 80 ladder proteins. Details of each plasmid construction are provided in Supplementary Note 3.

### Expression

Individual and coexpressed protein expression vectors were transformed into competent BL21(DE3)pLysS cells. Three to five colonies from a fresh transformation plate were inoculated into 100 ml 2×TY media containing 50 µg/ml ampicillin and 25 µg/ml chloramphenicol, incubated at 37 °C in a shaking incubator until the OD_600_ was between 0.1 and 0.15, transferred to a 21 °C shaking incubator, and induced with 0.2 mM IPTG when the OD_600_ was between 0.5 and 0.8. The culture was harvested 15 to 18 h later by centrifuging in two 50 ml Falcon tubes in a tabletop centrifuge at 3000 g for 10 min at room temperature. The supernatant was discarded and each pellet resuspended in 8 ml P300 (50 mM sodium phosphate pH 7.0, 300 mM NaCl, 1 mM benzamidine, 5 mM 2-mercaptoethanol) before flash freezing in liquid nitrogen and storage at − 20 °C.

### Purification

The thawed resuspended aliquot from ~ 50 ml of culture was sonicated in a Branson S-450D sonicator for 10 s at 40% maximum power, 50% cycle, cooled on ice for 20 s and the sonication repeated. 1.3 ml of sonicated extract was aliquoted into each of four 1.5 ml microcentrifuge tubes and centrifuged at maximum speed (13 K rpm) for 3 min at room temperature. The supernatant (~ 5 ml) was transferred to a 15 ml Falcon tube containing 0.5 ml of Talon Superflow metal affinity resin (Clontech, 635669) equilibrated in P300 buffer and incubated on a rotator for 20 min at room temperature. The pellet from one of the 1.5 ml microcentrifuge tubes was centrifuged for 5 s to gather any supernatant left on the sides of the tube. This residual supernatant was discarded and the remaining pellet resuspended in a total of 1.3 ml P300 solution.

The incubated Talon resin was sedimented in a tabletop centrifuge at 700*g* for 5 min at room temperature, and the supernatant saved as the Talon flow through (FT). The Talon resin was then washed twice with 10 ml of P300 buffer by mixing the resin with the buffer, centrifuging as before at 700*g* for 5 min at room temperature and pouring off the supernatant. The washed resin was resuspended in 3 ml P300 buffer before transferring to a disposable BioSpin column (Bio-Rad, 732-6008) clamped to a retort stand and allowing to drain. Samples were eluted from the Talon resin by adding 0.5 ml P300 + 200 mM imidazole to the top of resin and collecting the eluted material into a 1.5 ml microcentrifuge tube. Four such fractions were collected.

### Gel electrophoresis

Protein samples were boiled for 1 to 2 min or heated to 95 °C for 3 to 5 min in a modified Laemmli sample loading buffer (1 × sample loading buffer contains 62.5 mM Bis–Tris pH 7.0, 10% glycerol, 2% SDS, 7.5% 2-mercaptoethanol, 0.02% bromophenol blue). Samples were electrophoresed in BioRad Mini-PROTEAN gel electrophoresis units in protein gel running buffer (50 mM Tris base, 0.38 M glycine, 0.1% SDS).

### Western blotting

SDS-PAGE electrophoresis gels were blotted onto Protran 0.2 NC nitrocellulose membrane (Cytiva 10600009) using the standard SD program (25 V, 1.0 A, 30 min) on a Bio-Rad Trans-Blot semi-dry blotting device (Bio-Rad 1700-1918). The blot was blocked using 2% nonfat dry milk before incubating with 1:5000 dilution of mouse IgG kappa binding protein HRP conjugate (Santa Cruz sc-516102) or 1:5000 dilution of HRP-linked donkey anti-rabbit IgG (Cytiva NA934-1ML). The blot was visualized using SuperSignal West Pico PLUS chemiluminescent substrate (ThermoFisher 34580) on X-ray film (RPI 248304).

## Supplementary Information

Supplementary Information.
