# Supplementary material for: The Penn State Protein Ladder system for inexpensive protein molecular weight markers
Source: Sci Rep. 2021 Aug 18;11:16703. doi: 10.1038/s41598-021-96051-x (PMC8373980; doi:10.1038/s41598-021-96051-x)
Supplement: Supplementary file 1 — Supplementary Information. [file 41598_2021_96051_MOESM1_ESM.pdf]

The Penn State Protein Ladder System for inexpensive protein molecular weight markers

Supplementary Information

Ryan T. Santilli, John E. Williamson III, Yoshitaka Shibata,

Rosalie P. Sowers ,Andrew N. Fleischman and Song Tan

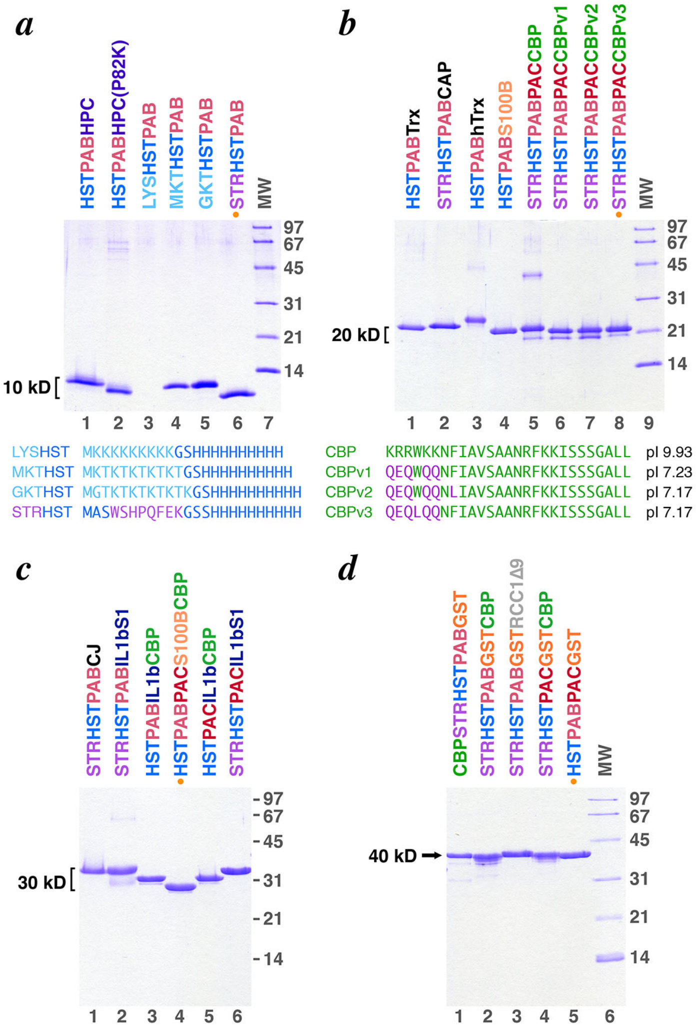

Supplementary Figure 1: Mobility on SDS-PAGE of different 10, 20, 30 and 40 kD ladder proteins candidates purified by metal affinity chromatography.

The protein selected for the Penn State ladder for each molecular weight is indicated by an orange dot above each gel.

(a) Comparison of 10 kD candidate proteins. The HSTPABHPC, HSTPABHPC(P82K), LYSHSTPAB, MKTHSTPAB, GKTHSTPAB and STRHSTPAB proteins are shown in lanes 1 through 6 respectively. The N-terminal sequence of the LYSHST, MKTHST, GKTHST and STRHST regions of the proteins are shown below the gel.

(b) Comparison of the 20 kD HSTPABTrx, STRHSTPABCAP, HSTPABhTrx, HSTPABS100B, STRHSTPABPACCBP, STRHSTPABPACCBPv1, STRHSTPABPACCBPv2 and STRHSTPABPACCBPv3 proteins are shown in lanes 1 through 8 respectively. The C-terminal sequences and the isoelectric point for the STRHSTPABPACCBP, STRHSTPABPACCBPv1, STRHSTPABPACCBPv2 and STRHSTPABPACCBPv3 proteins are shown below the gel.

(c) Comparison of 30 kD candidate proteins. The STRHSTPABCJ, STRHSTPABIL1bS1, STRHSTPABIL1bCBP, HSTPABPACS100BCBP, HSTPACIL1bCBP, STRHSTPACIL1bS1 proteins are shown in lanes 1 through 6.

(d) Comparison of 40 kD candidate proteins. The CBPSTRHSTPABGST, STRHSTPABGSTCBP, STRHSTPABGSTdRCC1Δ9, STRHSTPACGSTCBP and HSTPABPACGST proteins are shown in lanes 1 through 5.

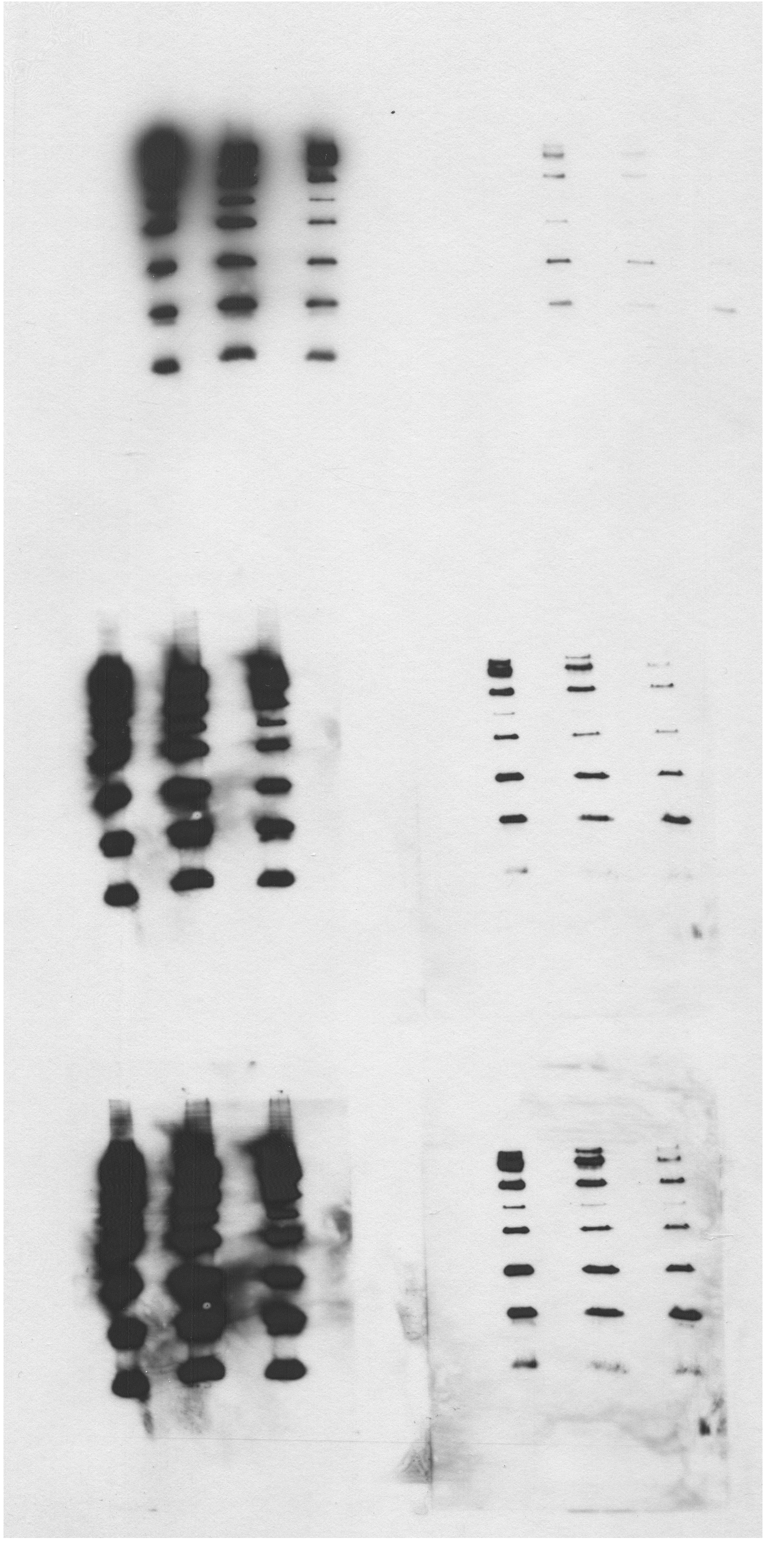

Supplementary Figure 2: Uncropped, full length blot source for Figure 5.

Supplementary Note 1

Anomalous mobility of candidate 10, 20, 30 and 40 kD ladder proteins

We found that many of the proteins we originally tested migrated anomalously slowly on SDS-PAGE, as judged in comparison to the Bio-Rad LMW (low molecular weight) markers. This was particularly noticeable for the lower molecular weight (10-30 kD) ladder proteins. When we observed our first HSTPABHPC (10xHIS-IgG binding domain-heavy chain of Protein C) 10 kD candidate protein construct migrated anomalously slowly, we examined the effect of changing the proline residue in the HPC tag. Mutating the HPC proline residue to lysine did increase the mobility, but this mutated protein still appeared to migrate anomalously slowly (**Suppl. Fig. 1a** lanes 1-2). To explore if we could improve the mobility and the expression level, we replaced the C-terminal HPC peptide with an N-terminal stretch of lysine residues (all coded by AAA codons and unlikely to form stable mRNA secondary structure, LYSHSTPAB construct) or alternating lysine-threonine residues (coded by AAA and ACA codons, MKTHSTPAB construct) or an MKTHSTPAB variant replacing the first lysine residue with glycine (GKTHSTPAB). The LYSHSTPAB construct with 9 consecutive lysine residues immediately following the START codon expressed very poorly, if at all, whereas the MKTHSTPAB and GKTHSTPAB residues expressed well but with slightly decreased mobility compared to HSTPABHPC (**Suppl. Fig. 1a** lanes 3-5). We considered whether the N-end rule for protein stability might account for the reduced expression of the LYSHSTPAB construct, but the high level expression of the MKTHSTPAB which also contains a lysine residue at position 2 would argue against this. The best 10 kD construct was STRHSTPAB, containing the Strep peptide followed by the histidine tag and the IgG binding domain B (**Suppl. Fig 1a** lane 6).

The 20 and 30 kD proteins were equally or more problematic. The 20 kD construct containing the S100 calcium binding protein B (S100B) migrated closer to the expected position than constructs containing thioredoxin (Trx) and the cytoplasmic linker protein 170 cytoskeleton-associated protein glycine-rich domain (CAP)[1] (compare **Suppl. Fig.**

1b lanes 1-4). However, to increase the Western blotting signal (see below), we reengineered most constructs to include tandem IgG binding domains (Protein A IgG binding domains B and C, abbreviated as PABPAC). The resulting 20 kD STR-HST-PAB-PAC-CBP construct containing a calmodulin-binding peptide tag (CBP) did not transfer well using Towbin Western blotting buffer (data not shown). We hypothesized that the high pI of 9.93 for this construct due to the CBP tag contributed to the poor Western transfer and we therefore engineered a version to remove or change the charge of CBP tag basic residues. The resulting protein, STRHSTPABPACCBPv1, did transfer efficiently by Western blotting but suffered from partial degradation to a slightly truncated product. Since the truncated product was purified by metal affinity chromatography via a tag near the N-terminus, we surmised the protein was degraded from the C-terminus. Based on the molecular weight of the truncated product, we conjectured the truncation was due to chymotrypsin-like proteolysis near a tryptophan or phenylalanine residue and consequently mutated these residues individually. Significantly less truncated product was observed for the STRHSTPABPACCBPv3 construct, although some truncated product remained (**Suppl. Fig. 1b** lane 8). Including protease inhibitors such as PMSF further reduced the amount of truncated product (data not shown). For the 30 kD protein, a series of constructs containing the *Campylobacter jejuni* putative periplasmic polyisoprenoid-binding protein (CJ)[2], the interleukin 1 beta (IL1b) protein with or without an engineered metal binding loop[3,4], or the S100B protein were expressed with the HST-PAB-PAC-S100B-CBP construct coming closest to the expected migration on SDS-PAGE (**Suppl. Fig. 1c**). The 40 kD proteins we expressed all contained glutathione S-transferase (GST) and migrated mostly as expected. We did not observe significant mobility differences installing the CBP tag at the N- or C-terminus or replacing the IgG binding domain B with domain C (**Suppl. Fig. 1d**).

References for Supplementary Notes

1. Mishima, M. *et al.* Structural basis for tubulin recognition by cytoplasmic linker protein 170 and its autoinhibition. *Proc Natl Acad Sci USA* **104,** 10346–10351 (2007).

2. Huber, T. R., McPherson, E. C., Keating, C. E. & Snow, C. D. Installing Guest Molecules at Specific Sites within Scaffold Protein Crystals. *Bioconjug Chem* acs.bioconjchem.7b00668 (2017). doi:10.1021/acs.bioconjchem.7b00668

3. Tocci, M. J. *et al.* Expression in Escherichia coli of fully active recombinant human IL 1 beta: comparison with native human IL 1 beta. *J. Immunol.* **138,** 1109–1114 (1987).

4. Barthelmes, K. *et al.* Engineering encodable lanthanide-binding tags into loop regions of proteins. *J Am Chem Soc* **133,** 808–819 (2011).

5. Tan, S., Kern, R. C. & Selleck, W. The pST44 polycistronic expression system for producing protein complexes in Escherichia coli. *Protein Expr Purif* **40,** 385–395 (2005).

6. Mandecki, W., Hayden, M. A., Shallcross, M. A. & Stotland, E. A totally synthetic plasmid for general cloning, gene expression and mutagenesis in Escherichia coli. *Gene* **94,** 103–107 (1990).

Supplementary Note 2

Penn State Ladder protein sequences

1. 10 kD STRHSTPAB 10.016 kDa pI 7.19 89 aa

MASWSHPQFEKGSSHHHHHHHHHHSSGSGSADNKFNKEQQNAFYEILHLPNLNEEQRNGFIQSLKDDPSQSANLLAEAKKLNDAQA
PKGS

2. 15 kD HSTPABPAC 15.012 kDa pI 6.65 133 aa

MGSHHHHHHHHHHGSADNKFNKEQQNAFYEILHLPNLNEEQRNGFIQSLKDDPSQSANLLAEAKKLNDAQAPKGSADNKFNKEQQN
AFYEILHLPNLTEEQRNGFIQSLKDDPSVSKEILAEAKKLNDAQAPAG

3. 20 kD STRHSTPABPACCBPv3 19.877 kDa pI 7.17 179 aa

MASWSHPQFEKGSSHHHHHHHHHHSSGSGSADNKFNKEQQNAFYEILHLPNLNEEQRNGFIQSLKDDPSQSANLLAEAKKLNDAQA
PKGSADNKFNKEQQNAFYEILHLPNLTEEQRNGFIQSLKDDPSVSKEILAEAKKLNDAQAPKGSGAGQEQLQQNFIAVSAANRFKK
ISSSGALL

4. 30 kD HSTPABPACS100BCBP 29.773 kDa pI 6.26 267 aa

MGSHHHHHHHHHHGSADNKFNKEQQNAFYEILHLPNLNEEQRNGFIQSLKDDPSQSANLLAEAKKLNDAQAPKGSADNKFNKEQQN
AFYEILHLPNLTEEQRNGFIQSLKDDPSVSKEILAEAKKLNDAQAPAGGSGSGSGSGSGSGSSELEKAMVALIDVFHQYSGREGDK
HKLKKSELKELINNELSHFLEEIKEQEVVDKVMETLDNDGDGECDFQEFMAFVAMVTTACHEFFEHEAGKRRWKKNFIAVSAANRF
KKISSSGALL

5. 40 kD HSTPABPACGST 40.362 kDa pI 6.60 350 aa

MGSHHHHHHHHHHGSADNKFNKEQQNAFYEILHLPNLNEEQRNGFIQSLKDDPSQSANLLAEAKKLNDAQAPKGSADNKFNKEQQN
AFYEILHLPNLTEEQRNGFIQSLKDDPSVSKEILAEAKKLNDAQAPAGSPILGYWKIKGLVQPTRLLLEYLEEKYEEHLYERDEGD
KWRNKKFELGLEFPNLPYYIDGDVKLTQSMAIIRYIADKHNMLGGCPKERAEISMLEGAVLDIRYGVSRIAYSKDFETLKVDFLSK
LPEMLKMFEDRLCHKTYLNGDHVTHPDFMLYDALDVVLYMDPMCLDAFPKLVCFKKRIEAIPQIDKYLKSSKYIAWPLQGWQATFG
GGDHPPK

6. 50 kD STRHSTPABMBP 50.310 kDa pI 6.00 456 aa

MASWSHPQFEKGSSHHHHHHHHHHSSGSGSADNKFNKEQQNAFYEILHLPNLNEEQRNGFIQSLKDDPSQSANLLAEAKKLNDAQA
PKGSKIEEGKLVIWINGDKGYNGLAEVGKKFEKDTGIKVTVEHPDKLEEKFPQVAATGDGPDIIFWAHDRFGGYAQSGLLAEITPD
KAFQDKLYPFTWDAVRYNGKLIAYPIAVEALSLIYNKDLLPNPPKTWEEIPALDKELKAKGKSALMFNLQEPYFTWPLIAADGGYA
FKYENGKYDIKDVGVDNAGAKAGLTFLVDLIKNKHMNADTDYSIAEAAFNKGETAMTINGPWAWSNIDTSKVNYGVTVLPTFKGQP
SKPFVGVLSAGINAASPNKELAKEFLENYLLTDEGLEAVNKDKPLGAVALKSYEEELAKDPRIAATMENAQKGEIMPNIPQMSAFW
YAVRTAVINAASGRQTVDEALKDAQTN

7. 60 kD HSTPABPACdRCC1 60.029 kDa pI 6.93 554 aa

MGSHHHHHHHHHHGSADNKFNKEQQNAFYEILHLPNLNEEQRNGFIQSLKDDPSQSANLLAEAKKLNDAQAPKGSADNKFNKEQQN
AFYEILHLPNLTEEQRNGFIQSLKDDPSVSKEILAEAKKLNDAQAPAGRKALTNNNNAGEAEQQPPKAKRARIAFHLELPKRRTVL
GNVLVCGNGDVGQLGLGEDILERKRLSPVAGIPDAVDISAGGMHNLVLTKSGDIYSFGCNDEGALGRDTSEDGSESKPDLIDLPGK
ALCISAGDSHSACLLEDGRVFAWGSFRDSHGNMGLTIDGNKRTPIDLMEGTVCCSIASGADHLVILTTAGKVFTVGCAEQGQLGRL
SERSISGEGRRGKRDLLRPTQLIITRAKPFEAIWATNYCTFMRESQTQVIWATGLNNFKQLAHETKGKEFALTPIKTELKDIRHIA
GGQHHTVILTTDLKCSVVGRPEYGRLGLGDVKDVVEKPTIVKKLTEKIVSVGCGEVCSYAVTIDGKLYSWGSGVNNQLGVGDGDDE
LEPIVVVSKNTQGKHMLLASGGGQHAIFLVKADKQDQAG

8. 80 kD STRHSTPABPACIL1bdRCC1 79.830 kDa pI 6.97 732 aa

MASWSHPQFEKGSSHHHHHHHHHHSSGSGSADNKFNKEQQNAFYEILHLPNLNEEQRNGFIQSLKDDPSQSANLLAEAKKLNDAQA
PKGSADNKFNKEQQNAFYEILHLPNLTEEQRNGFIQSLKDDPSVSKEILAEAKKLNDAQAPKGSGSSGSSGSSAPVRSLNCTLRDS
QQKSLVMSGPYELKALHLQGQDMEQQVVFSMSFVQGEESNDKIPVALGLKEKNLYLSCVLKDDKPTLQLESVDPKNYPKKKMEKRF
VFNKIEINNKLEFESAQFPNWYISTSQAENMPVFLGGTKGGQDITDFTMQFVSSAGRKALTNNNNAGEAEQQPPKAKRARIAFHLE
LPKRRTVLGNVLVCGNGDVGQLGLGEDILERKRLSPVAGIPDAVDISAGGMHNLVLTKSGDIYSFGCNDEGALGRDTSEDGSESKP
DLIDLPGKALCISAGDSHSACLLEDGRVFAWGSFRDSHGNMGLTIDGNKRTPIDLMEGTVCCSIASGADHLVILTTAGKVFTVGCA
EQGQLGRLSERSISGEGRRGKRDLLRPTQLIITRAKPFEAIWATNYCTFMRESQTQVIWATGLNNFKQLAHETKGKEFALTPIKTE
LKDIRHIAGGQHHTVILTTDLKCSVVGRPEYGRLGLGDVKDVVEKPTIVKKLTEKIVSVGCGEVCSYAVTIDGKLYSWGSGVNNQL
GVGDGDDELEPIVVVSKNTQGKHMLLASGGGQHAIFLVKADKQDQ

9. 100 kD STRHSTPABPACIL1bQRS 98.269 kDa pI 6.46 866 aa

MASWSHPQFEKGSSHHHHHHHHHHSSGSGSADNKFNKEQQNAFYEILHLPNLNEEQRNGFIQSLKDDPSQSANLLAEAKKLNDAQA
PKGSADNKFNKEQQNAFYEILHLPNLTEEQRNGFIQSLKDDPSVSKEILAEAKKLNDAQAPKGSGSSGSSGSSAPVRSLNCTLRDS
QQKSLVMSGPYELKALHLQGQDMEQQVVFSMSFVQGEESNDKIPVALGLKEKNLYLSCVLKDDKPTLQLESVDPKNYPKKKMEKRF
VFNKIEINNKLEFESAQFPNWYISTSQAENMPVFLGGTKGGQDITDFTMQFVSSAGSEAEARPTNFIRQIIDEDLASGKHTTVHTR
FPPEPNGYLHIGHAKSICLNFGIAQDYKGQCNLRFDDTNPVKEDIEYVESIKNDVEWLGFHWSGNVRYSSDYFDQLHAYAIELINK
GLAYVDELTPEQIREYRGTLTQPGKNSPYRDRSVEENLALFEKMRAGGFEEGKACLRAKIDMASPFIVMRDPVLYRIKFAEHHQTG
NKWCIYPMYDFTHCISDALEGITHSLCTLEFQDNRRLYDWVLDNITIPVHPRQYEFSRLNLEYTVMSKRKLNLLVTDKHVEGWDDP
RMPTISGLRRRGYTAASIREFCKRIGVTKQDNTIEMASLESCIREDLNENAPRAMAVIDPVKLVIENYQGEGEMVTMPNHPNKPEM
GSRQVPFSGEIWIDRADFREEANKQYKRLVLGKEVRLRNAYVIKAERVEKDAEGNITTIFCTYDADTLSKDPADGRKVKGVIHWVS
AAHALPVEIRLYDRLFSVPNPGAADDFLSVINPESLVIKQGFAEPSLKDAVAGKAFQFEREGYFCLDSRHSTAEKPVFNRTVGLRD
TWAKVGE

10. 150 kD STRHSTPABMBPpepN 149.946 kDa pI 5.25 1333 aa

MASWSHPQFEKGSSHHHHHHHHHHSSGSGSADNKFNKEQQNAFYEILHLPNLNEEQRNGFIQSLKDDPSQSANLLAEAKKLNDAQA
PKGSKIEEGKLVIWINGDKGYNGLAEVGKKFEKDTGIKVTVEHPDKLEEKFPQVAATGDGPDIIFWAHDRFGGYAQSGLLAEITPD
KAFQDKLYPFTWDAVRYNGKLIAYPIAVEALSLIYNKDLLPNPPKTWEEIPALDKELKAKGKSALMFNLQEPYFTWPLIAADGGYA
FKYENGKYDIKDVGVDNAGAKAGLTFLVDLIKNKHMNADTDYSIAEAAFNKGETAMTINGPWAWSNIDTSKVNYGVTVLPTFKGQP
SKPFVGVLSAGINAASPNKELAKEFLENYLLTDEGLEAVNKDKPLGAVALKSYEEELAKDPRIAATMENAQKGEIMPNIPQMSAFW
YAVRTAVINAASGRQTVDEALKDAQTNSGSTQQPQAKYRHDYRAPDYQITDIDLTFDLDAQKTVVTAVSQAVRHGASDAPLRLNGE
DLKLVSVHINDEPWTAWKEEEGALVISNLPERFTLKIINEISPAANTALEGLYQSGDALCTQCEAEGFRHITYYLDRPDVLARFTT
KIIADKIKYPFLLSNGNRVAQGELENGRHWVQWQDPFPKPCYLFALVAGDFDVLRDTFTTRSGREVALELYVDRGNLDRAPWAMTS
LKNSMKWDEERFGLEYDLDIYMIVAVDFFNMGAMENKGLNIFNSKYVLARTDTATDKDYLDIERVIGHEYFHNWTGNRVTCRDWFQ
LSLKEGLTVFRDQEFSSDLGSRAVNRINNVRTMRGLQFAEDASPMAHPIRPDMVIEMNNFYTLTVYEKGAEVIRMIHTLLGEENFQ
KGMQLYFERHDGSAATCDDFVQAMEDASNVDLSHFRRWYSQSGTPIVTVKDDYNPETEQYTLTISQRTPATPDQAEKQPLHIPFAI
ELYDNEGKVIPLQKGGHPVNSVLNVTQAEQTFVFDNVYFQPVPALLCEFSAPVKLEYKWSDQQLTFLMRHARNDFSRWDAAQSLLA
TYIKLNVARHQQGQPLSLPVHVADAFRAVLLDEKIDPALAAEILTLPSVNEMAELFDIIDPIAIAEVREALTRTLATELADELLAI
YNANYQSEYRVEHEDIAKRTLRNACLRFLAFGETHLADVLVSKQFHEANNMTDALAALSAAVAAQLPCRDALMQEYDDKWHQNGLV
MDKWFILQATSPAANVLETVRGLLQHRSFTMSNPNRIRSLIGAFAGSNPAAFHAEDGSGYLFLVEMLTDLNSRNPQVASRLIEPLI
RLKRYDAKRQEKMRAALEQLKGLENLSGDLYEKITKALAQYTKN

11. 250 kD STRHSTPABPACIL1bQRSSTRHSTPABMBPpepN
249.895 kDa pI 5.76 2216 aa

MASWSHPQFEKGSSHHHHHHHHHHSSGSGSADNKFNKEQQNAFYEILHLPNLNEEQRNGFIQSLKDDPSQSANLLAEAKKLNDAQA
PKGSADNKFNKEQQNAFYEILHLPNLTEEQRNGFIQSLKDDPSVSKEILAEAKKLNDAQAPKGSGSSGSSGSSAPVRSLNCTLRDS
QQKSLVMSGPYELKALHLQGQDMEQQVVFSMSFVQGEESNDKIPVALGLKEKNLYLSCVLKDDKPTLQLESVDPKNYPKKKMEKRF
VFNKIEINNKLEFESAQFPNWYISTSQAENMPVFLGGTKGGQDITDFTMQFVSSAGEGQGQGQGEGSGSEAEARPTNFIRQIIDED
LASGKHTTVHTRFPPEPNGYLHIGHAKSICLNFGIAQDYKGQCNLRFDDTNPVKEDIEYVESIKNDVEWLGFHWSGNVRYSSDYFD
QLHAYAIELINKGLAYVDELTPEQIREYRGTLTQPGKNSPYRDRSVEENLALFEKMRAGGFEEGKACLRAKIDMASPFIVMRDPVL
YRIKFAEHHQTGNKWCIYPMYDFTHCISDALEGITHSLCTLEFQDNRRLYDWVLDNITIPVHPRQYEFSRLNLEYTVMSKRKLNLL
VTDKHVEGWDDPRMPTISGLRRRGYTAASIREFCKRIGVTKQDNTIEMASLESCIREDLNENAPRAMAVIDPVKLVIENYQGEGEM
VTMPNHPNKPEMGSRQVPFSGEIWIDRADFREEANKQYKRLVLGKEVRLRNAYVIKAERVEKDAEGNITTIFCTYDADTLSKDPAD
GRKVKGVIHWVSAAHALPVEIRLYDRLFSVPNPGAADDFLSVINPESLVIKQGFAEPSLKDAVAGKAFQFEREGYFCLDSRHSTAE
KPVFNRTVGLRDTWAKVGERPMASWSHPQFEKGSSHHHHHHHHHHSSGSGSADNKFNKEQQNAFYEILHLPNLNEEQRNGFIQSLK
DDPSQSANLLAEAKKLNDAQAPKGSKIEEGKLVIWINGDKGYNGLAEVGKKFEKDTGIKVTVEHPDKLEEKFPQVAATGDGPDIIF
WAHDRFGGYAQSGLLAEITPDKAFQDKLYPFTWDAVRYNGKLIAYPIAVEALSLIYNKDLLPNPPKTWEEIPALDKELKAKGKSAL
MFNLQEPYFTWPLIAADGGYAFKYENGKYDIKDVGVDNAGAKAGLTFLVDLIKNKHMNADTDYSIAEAAFNKGETAMTINGPWAWS
NIDTSKVNYGVTVLPTFKGQPSKPFVGVLSAGINAASPNKELAKEFLENYLLTDEGLEAVNKDKPLGAVALKSYEEELAKDPRIAA
TMENAQKGEIMPNIPQMSAFWYAVRTAVINAASGRQTVDEALKDAQTNSGSTQQPQAKYRHDYRAPDYQITDIDLTFDLDAQKTVV
TAVSQAVRHGASDAPLRLNGEDLKLVSVHINDEPWTAWKEEEGALVISNLPERFTLKIINEISPAANTALEGLYQSGDALCTQCEA
EGFRHITYYLDRPDVLARFTTKIIADKIKYPFLLSNGNRVAQGELENGRHWVQWQDPFPKPCYLFALVAGDFDVLRDTFTTRSGRE
VALELYVDRGNLDRAPWAMTSLKNSMKWDEERFGLEYDLDIYMIVAVDFFNMGAMENKGLNIFNSKYVLARTDTATDKDYLDIERV
IGHEYFHNWTGNRVTCRDWFQLSLKEGLTVFRDQEFSSDLGSRAVNRINNVRTMRGLQFAEDASPMAHPIRPDMVIEMNNFYTLTV
YEKGAEVIRMIHTLLGEENFQKGMQLYFERHDGSAATCDDFVQAMEDASNVDLSHFRRWYSQSGTPIVTVKDDYNPETEQYTLTIS
QRTPATPDQAEKQPLHIPFAIELYDNEGKVIPLQKGGHPVNSVLNVTQAEQTFVFDNVYFQPVPALLCEFSAPVKLEYKWSDQQLT
FLMRHARNDFSRWDAAQSLLATYIKLNVARHQQGQPLSLPVHVADAFRAVLLDEKIDPALAAEILTLPSVNEMAELFDIIDPIAIA
EVREALTRTLATELADELLAIYNANYQSEYRVEHEDIAKRTLRNACLRFLAFGETHLADVLVSKQFHEANNMTDALAALSAAVAAQ
LPCRDALMQEYDDKWHQNGLVMDKWFILQATSPAANVLETVRGLLQHRSFTMSNPNRIRSLIGAFAGSNPAAFHAEDGSGYLFLVE
MLTDLNSRNPQVASRLIEPLIRLKRYDAKRQEKMRAALEQLKGLENLSGDLYEKITKALAQYTKNLQ

10 kD candidate proteins

1. HSTPABHPC 9.984 kDa pI 6.72 89 aa

MGSHHHHHHHHHHGSADNKFNKEQQNAFYEILHLPNLNEEQRNGFIQSLKDDPSQSANLLAEAKKLNDAQAPKGSSEDQVDPRLID
GKAG

2. HSTPABHPC(P82K) 10.015 kDa pI 6.89 89 aa

MGSHHHHHHHHHHGSADNKFNKEQQNAFYEILHLPNLNEEQRNGFIQSLKDDPSQSANLLAEAKKLNDAQAPKGSSEDQVDKRLID
GKAG

3. LYSHSTPAB 10.005 kDa pI 10.53 88 aa

MKKKKKKKKKGSHHHHHHHHHHSSGSGSADNKFNKEQQNAFYEILHLPNLNEEQRNGFIQSLKDDPSQSANLLAEAKKLNDAQAPK
GS

4. MKTHSTPAB 9.998 kDa pI 10.02 89 aa

MKTKTKTKTKTGSHHHHHHHHHHSSGSGSADNKFNKEQQNAFYEILHLPNLNEEQRNGFIQSLKDDPSQSANLLAEAKKLNDAQAP
KGS

5. GKTHSTPAB 10.011 kDa pI 10.02 90 aa

MGTKTKTKTKTKGSSHHHHHHHHHHSSGSGSADNKFNKEQQNAFYEILHLPNLNEEQRNGFIQSLKDDPSQSANLLAEAKKLNDAQ
APKGS

6. STRHSTPAB 10.016 kDa pI 7.19 89 aa

MASWSHPQFEKGSSHHHHHHHHHHSSGSGSADNKFNKEQQNAFYEILHLPNLNEEQRNGFIQSLKDDPSQSANLLAEAKKLNDAQA
PKGS

20 kD candidate proteins

1. HSTPABTrx 19.972 kDa pI 6.37 181 aa

MGSHHHHHHHHHHGSADNKFNKEQQNAFYEILHLPNLNEEQRNGFIQSLKDDPSQSANLLAEAKKLNDAQAPKGSDKIIHLTDDSF
DTDVLKADGAILVDFWAEWCGPCKMIAPILDEIADEYQGKLTVAKLNIDQNPGTAPKYGIRGIPTLLLFKNGEVAATKVGALSKGQ
LKEFLDANLA

2. STRHSTPABCAP 19.996 kDa pI 9.13 182 aa

MASWSHPQFEKGSSHHHHHHHHHHSSGSGSADNKFNKEQQNAFYEILHLPNLNEEQRNGFIQSLKDDPSQSANLLAEAKKLNDAQA
PKGSGERELKIGDRVLVGGTKAGVVRFLGETDFAKGEWCGVELDEPLGKNDGAVAGTRYFQCQPKYGLFAPVHKVTKIGFPSTTPA
KAKANAVRRVM

3. HSTPABhTrx 20.252 kDa pI 6.39 181 aa

MGSHHHHHHHHHHGSADNKFNKEQQNAFYEILHLPNLNEEQRNGFIQSLKDDPSQSANLLAEAKKLNDAQAPKGSTTFNIQDGPDF
QDRVVNSETPVVVDFHAQWCGPCKILGPRLEKMVAKQHGKVVMAKVDIDDHTDLAIEYEVSAVPTVLAMKNGDVVDKFVGIKDEDQ
LEAFLKKLIG

4. HSTPABS100B 19.975 kDa pI 5.43 179 aa

MGSHHHHHHHHHHGSADNKFNKEQQNAFYEILHLPNLNEEQRNGFIQSLKDDPSQSANLLAEAKKLNDAQAPKGSGSGSGSGSGSG
SGSSELEKAMVALIDVFHQYSGREGDKHKLKKSELKELINNELSHFLEEIKEQEVVDKVMETLDNDGDGECDFQEFMAFVAMVTTA
CHEFFEHE

5. STRHSTPABPACCBP 20.005 kDa pI 9.93 179 aa

MASWSHPQFEKGSSHHHHHHHHHHSSGSGSADNKFNKEQQNAFYEILHLPNLNEEQRNGFIQSLKDDPSQSANLLAEAKKLNDAQA
PKGSADNKFNKEQQNAFYEILHLPNLTEEQRNGFIQSLKDDPSVSKEILAEAKKLNDAQAPKGSGAGKRRWKKNFIAVSAANRFKK
ISSSGALL

6. STRHSTPABPACCBPv1 19.950 kDa pI 7.23 179 aa

MASWSHPQFEKGSSHHHHHHHHHHSSGSGSADNKFNKEQQNAFYEILHLPNLNEEQRNGFIQSLKDDPSQSANLLAEAKKLNDAQA
PKGSADNKFNKEQQNAFYEILHLPNLTEEQRNGFIQSLKDDPSVSKEILAEAKKLNDAQAPKGSGAGQEQWQQNFIAVSAANRFKK
ISSSGALL

7. STRHSTPABPACCBPv2 19.916 kDa pI 7.17 179 aa

MASWSHPQFEKGSSHHHHHHHHHHSSGSGSADNKFNKEQQNAFYEILHLPNLNEEQRNGFIQSLKDDPSQSANLLAEAKKLNDAQA
PKGSADNKFNKEQQNAFYEILHLPNLTEEQRNGFIQSLKDDPSVSKEILAEAKKLNDAQAPKGSGAGQEQWQQNLIAVSAANRFKK
ISSSGALL

8. STRHSTPABPACCBPv3 19.877 kDa pI 7.17 179 aa

MASWSHPQFEKGSSHHHHHHHHHHSSGSGSADNKFNKEQQNAFYEILHLPNLNEEQRNGFIQSLKDDPSQSANLLAEAKKLNDAQA
PKGSADNKFNKEQQNAFYEILHLPNLTEEQRNGFIQSLKDDPSVSKEILAEAKKLNDAQAPKGSGAGQEQLQQNFIAVSAANRFKK
ISSSGALL

30 kD candidate proteins

1. STRHSTPABCJ 29.997 kDa pI 7.30 271 aa

MASWSHPQFEKGSSHHHHHHHHHHSSGSGSADNKFNKEQQNAFYEILHLPNLNEEQRNGFIQSLKDDPSQSANLLAEAKKLNDAQA
PKGSGSEGQGQGQGESGSKEYTLDKAHTDVGFKIKHLQISNVKGNFKDYSAVIDFDPASAEFKKLDVTIKIASVNTENQTRNHLQQ
DDFFKAKKYPDMTFTMKKYEKIDNEKGKMTGTLTIAGVSKDIVLDAEIGGVAKGKDGKEIGFSLNGKIKRSDFKFATSTSTITLSD
DINLNIEVKANEKE

2. STRHSTPABIL1bS1 30.014 kDa pI 6.32 268 aa

MASWSHPQFEKGSSHHHHHHHHHHSSGSGSADNKFNKEQQNAFYEILHLPNLNEEQRNGFIQSLKDDPSQSANLLAEAKKLNDAQA
PKGSGSSGSSGSSAPVRSLNCTLRDSQQKSLVMSGPYELKALHLQGQDMEQQVVFSMSFVQGEESNGYIDTNNDGWIEGDELYDKI
PVALGLKEKNLYLSCVLKDDKPTLQLESVDPKNYPKKKMEKRFVFNKIEINNKLEFESAQFPNWYISTSQAENMPVFLGGTKGGQD
ITDFTMQFVSS

3. HSTPABIL1bCBP 29.642 kDa pI 9.51 265 aa

MGSHHHHHHHHHHGSADNKFNKEQQNAFYEILHLPNLNEEQRNGFIQSLKDDPSQSANLLAEAKKLNDAQAPKGSGSSGSSGSSAP
VRSLNCTLRDSQQKSLVMSGPYELKALHLQGQDMEQQVVFSMSFVQGEESNDKIPVALGLKEKNLYLSCVLKDDKPTLQLESVDPK
NYPKKKMEKRFVFNKIEINNKLEFESAQFPNWYISTSQAENMPVFLGGTKGGQDITDFTMQFVSSAGKRRWKKNFIAVSAANRFKK
ISSSGALL

4. HSTPABPACS100BCBP 29.773 kDa pI 6.26 267 aa

MGSHHHHHHHHHHGSADNKFNKEQQNAFYEILHLPNLNEEQRNGFIQSLKDDPSQSANLLAEAKKLNDAQAPKGSADNKFNKEQQN
AFYEILHLPNLTEEQRNGFIQSLKDDPSVSKEILAEAKKLNDAQAPAGGSGSGSGSGSGSSELEKAMVALIDVFHQYSGREGDK
HKLKKSELKELINNELSHFLEEIKEQEVVDKVMETLDNDGDGECDFQEFMAFVAMVTTACHEFFEHEAGKRRWKKNFIAVSAANRF
KKISSSGALL

5. HSTPACIL1bCBP 29.672 kDa pI 7.30 265 aa

MGSHHHHHHHHHHGSADNKFNKEQQNAFYEILHLPNLTEEQRNGFIQSLKDDPSVSKEILAEAKKLNDAQAPKGSGSSGSSGSSAP
VRSLNCTLRDSQQKSLVMSGPYELKALHLQGQDMEQQVVFSMSFVQGEESNDKIPVALGLKEKNLYLSCVLKDDKPTLQLESVDPK
NYPKKKMEKRFVFNKIEINNKLEFESAQFPNWYISTSQAENMPVFLGGTKGGQDITDFTMQFVSSAGKRRWKKNFIAVSAANRFKK
ISSSGALL

6. STRHSTPACIL1bS1 30.044 kDa pI 6.32 268 aa

MASWSHPQFEKGSSHHHHHHHHHHSSGSGSADNKFNKEQQNAFYEILHLPNLTEEQRNGFIQSLKDDPSVSKEILAEAKKLNDAQA
PKGSGSSGSSGSSAPVRSLNCTLRDSQQKSLVMSGPYELKALHLQGQDMEQQVVFSMSFVQGEESNGYIDTNNDGWIEGDELYDKI
PVALGLKEKNLYLSCVLKDDKPTLQLESVDPKNYPKKKMEKRFVFNKIEINNKLEFESAQFPNWYISTSQAENMPVFLGGTKGGQD
ITDFTMQFVSS

40 kD candidate proteins

1. CBPSTRHSTPABGST — 40.011 kDa — pI 8.06 — 351 aa

MSKRRWKKNFIAVSAANRFKKISSSGALLGSASWSHPQFEKGSSHHHHHHHHHHSSGSGSADNKFNKEQQNAFYEILHLPNLNEEQ
RNGFIQSLKDDPSQSANLLAEAKKLNDAQAPKGSSSPILGYWKIKGLVQPTRLLLEYLEEKYEEHLYERDEGDKWRNKKFELGLEF
PNLPYYIDGDVKLTQSMAIIRYIADKHNMLGGCPKERAEISMLEGAVLDIRYGVSRIAYSKDFETLKVDFLSKLPEMLKMFEDRLC
HKTYLNGDHVTHPDFMLYDALDVVLYMDPMCLDAFPKLVCFKKRIEAIPQIDKYLKSSKYIAWPLQGWQATFGGGDHPPKSDLSGG
GGENLYAG

2. STRHSTPABGSTCBP — 40.010 kDa — pI 8.06 — 351 aa

MASWSHPQFEKGSSHHHHHHHHHHSSGSGSADNKFNKEQQNAFYEILHLPNLNEEQRNGFIQSLKDDPSQSANLLAEAKKLNDAQA
PKGSSSPILGYWKIKGLVQPTRLLLEYLEEKYEEHLYERDEGDKWRNKKFELGLEFPNLPYYIDGDVKLTQSMAIIRYIADKHNML
GGCPKERAEISMLEGAVLDIRYGVSRIAYSKDFETLKVDFLSKLPEMLKMFEDRLCHKTYLNGDHVTHPDFMLYDALDVVLYMDPM
CLDAFPKLVCFKKRIEAIPQIDKYLKSSKYIAWPLQGWQATFGGGDHPPKSDLSGGGGENLYAGKRRWKKNFIAVSAANRFKKISS
SGALQSAG

3. STRHSTPABGSTRCC1Δ9 — 39.957 kDa — pI 7.05 — 352 aa

MASWSHPQFEKGSSHHHHHHHHHHSSGSGSADNKFNKEQQNAFYEILHLPNLNEEQRNGFIQSLKDDPSQSANLLAEAKKLNDAQA
PKGSSSPILGYWKIKGLVQPTRLLLEYLEEKYEEHLYERDEGDKWRNKKFELGLEFPNLPYYIDGDVKLTQSMAIIRYIADKHNML
GGCPKERAEISMLEGAVLDIRYGVSRIAYSKDFETLKVDFLSKLPEMLKMFEDRLCHKTYLNGDHVTHPDFMLYDALDVVLYMDPM
CLDAFPKLVCFKKRIEAIPQIDKYLKSSKYIAWPLQGWQATFGGGDHPPKSDLSGGGGENLYAGKALTNNNNAGEAEQQPPKAKRA
RIAQHLGAG

4. STRHSTPACGSTCBP — 40.040 kDa — pI 8.06 — 351 aa

MASWSHPQFEKGSSHHHHHHHHHHSSGSGSADNKFNKEQQNAFYEILHLPNLTEEQRNGFIQSLKDDPSVSKEILAEAKKLNDAQA
PKGSSSPILGYWKIKGLVQPTRLLLEYLEEKYEEHLYERDEGDKWRNKKFELGLEFPNLPYYIDGDVKLTQSMAIIRYIADKHNML
GGCPKERAEISMLEGAVLDIRYGVSRIAYSKDFETLKVDFLSKLPEMLKMFEDRLCHKTYLNGDHVTHPDFMLYDALDVVLYMDPM
CLDAFPKLVCFKKRIEAIPQIDKYLKSSKYIAWPLQGWQATFGGGDHPPKSDLSGGGGENLYAGKRRWKKNFIAVSAANRFKKISS
SGALQSAG

5. HSTPABPACGST — 40.362 kDa — pI 6.60 — 350 aa

MGSHHHHHHHHHHGSADNKFNKEQQNAFYEILHLPNLNEEQRNGFIQSLKDDPSQSANLLAEAKKLNDAQAPKGSADNKFNKEQQN
AFYEILHLPNLTEEQRNGFIQSLKDDPSVSKEILAEAKKLNDAQAPAGSPILGYWKIKGLVQPTRLLLEYLEEKYEEHLYERDEGD
KWRNKKFELGLEFPNLPYYIDGDVKLTQSMAIIRYIADKHNMLGGCPKERAEISMLEGAVLDIRYGVSRIAYSKDFETLKVDFLSK
LPEMLKMFEDRLCHKTYLNGDHVTHPDFMLYDALDVVLYMDPMCLDAFPKLVCFKKRIEAIPQIDKYLKSSKYIAWPLQGWQATFG
GGDHPPK

Supplementary Note 3

Plasmid construction

1. 10 kD STRHSTPAB
   pPSU10 (pST50Tr-STRHSTPAB)

The synthesized gBlock_STRHSTPAB_ENQ template was amplified using STO4889/STO4890 primers, digested with NdeI & BsrGI restriction endonucleases and subcloned into NdeI-BsrGI digested pST50Tr expression vector.

2. 15 kD HSTPABPAC
   pPSU15 (pST50Trc2-HSTPABPAC)

The gBlock_HSTPABPAC template was amplified using STO4891/STO4892 primers, digested with NdeI & BsrGI restriction endonucleases and subcloned into NdeI-BsrGI digested pST50Trc2 expression vector.

3. 20 kD STRHSTPABPACCBPv3
   pPSU20 (pST50Trc4-STRHSTPABPACCBPv3)

The BamHI-BsrGI GST cassette from pST50Tr-GSTNyEpl1Δ3HISx3 (S.T., unpublished) was subcloned into BamHI-BsrGI pST50Tr-STRHSTPAB to create pST50Tr-STRHSTPABGST. The NgoMIV-BsrGI CBP tag, amplified from the pST50Tr-CBPDHFR template[5] using STO5047/STO5048 primers, was subcloned into NgoMIV-BsrGI pST50Tr-STRHSTPABGST to produce the pST50Tr-STRHSTPABGSTCBP plasmid. The BglII-BsrGI PAC cassette, amplified from the gBlock_STRHSTPAC template using STO5066/STO5067 primers, was subcloned into BamHI-BsrGI pST50Tr-STRHSTPAB vector to create pST50Tr-STRHSTPABPAC. The NdeI-NgoMIV STRHSTPABPAC insert from pST50Tr-STRHSTPABPAC was subcloned into NdeI-NgoMIV pST50Tr-CBP vector prepared from the pST50Tr-STRHSTPABGSTCBP to produce the pST50Tr-STRHSTPABPACCBP plasmid. The NdeI-BsrGI STRHSTPABPACCBP cassette from pST50Tr-STRHSTPABPACCBP was subcloned into NdeI-BsrGI pST50Trc4 to produce pST50Trc4-STRHSTPABPACCBP in anticipation of subcloning into a pST44 polycistronic vector. The recoded NgoMIV-BsrGI CBPv1 tag mutating basic residues amplified from pST50Trc4-STRHSTPABPACCBP using the STO5113/STO314 primers was subcloned into NgoMIV-BsrGI pST50Trc4-STRHSTPABPACCBP to create pST50Trc4-STRHSTPABPACCBPv1. PCR based site-directed mutagenesis using STO5126/STO5127 primers was then used to mutate Trp157 to Leu in pST50Trc4-STRHSTPABPACCBPv1 to create the pST50Trc4-STRHSTPABPACCBPv3 plasmid.

4. 30 kD HSTPABPACS100BCBP
   pPSU30 (pST50Trc3-HSTPABPACS100BCBP)

The BamHI-BsrGI IL1b cassette derived from the gBlock_IL1bS1 template was subcloned into BamHI-BsrGI pST50Trc2-HSTPAB vector (this work) to create pST50Trc2-HSTPABIL1b. The NdeI-NgoMIV HSTPABIL1b cassette amplified from pST50Trc2-HSTPABIL1b using STO1099/STO5046 primers was subcloned into NdeI-NgoMIV pST50Tr-CBP from pST50-STRHSTPABGSTCBP vector to create pST50Tr-HSTPABIL1bCBP. The NdeI-BsrGI HSTPABIL1bCBP cassette from pST50Tr-HSTPABIL1bCBP was subcloned into NdeI-BsrGI

pST50Trc3 vector from pST50Trc3-STRHISNyEsa1x3 (S.T., unpublished) to create pST50Trc3-HSTPABIL1bCBP. The BamHI-BsrGI S100B coding region from gBlock_S100B_hTrx2 template amplified with STO5028/STO5029 primers was subcloned into BamHI-BsrGI pST50Trc2-HSTPAB vector to create pST50Trc2-HSTPABS100B. The BspEI-BsrGI S100B cassette amplified from pST50Trc2-HSTPABS100B template using STO5056/STO5057 primers was subcloned into NgoMIV-BsrGI pST50Trc2-HSTPABPAC vector to create pST50Trc2-HSTPAPPACS100B. The NdeI-NgoMIV HSTPABPACS100B cassette from pST50Trc2-HSTPABPACS100B was subcloned into NdeI-NgoMIV pST50Trc3-CBP from pST50Trc3-HSTPABIL1bCBP to create pST50Trc3-HSTPABPACS100BCBP.

5. 40 kD HSTPABPACGST
pPSU40 (pST50Trc2-HSTPABPACGST)

The NgoMIV-BsrGI GST cassette amplified from pET3a-GSTN (S.T., unpublished) using STO2300/STO5068 primers was subcloned into NgoMIV-BsrGI pST50Trc2-HSTPABPAC vector to create pST50Trc2-HSTPABPACGST.

6. 50 kD STRHSTPABMBP
pPSU50 (pST50Tr-STRHSTPABMBP)

The MBP cassette amplified from pST39-MBPHISDHFR[5] using STO2599/STO2600 primers was blunt-end ligated into phosphatased SmaI pWM529[6] to create pWM529-MBP. The BglII-BamHI MBP cassette from pWM529-MBP was subcloned into phosphatased BamHI pST50Tr-STRHSTPAB to create pST50Tr-STRHSTPABMBPv0. The internal BglII site in the MBP cassette was removed by silent PCR-based mutagenesis using STO5017/STO5018 primers to create pST50Tr-STRHSTPABMBPv1. The internal BspEI site in the MBP cassette was then removed by silent PCR-based mutagenesis using STO5019/STO5020 primers to create pST50Tr-STRHSTPABMBPv2. The terminal 3 codons in the MBP coding region were removed by amplifying the MBP cassette from pST50Tr-STRHSTPABMBPv2 (expresses 50.5 kD protein) using STO1099/STO5016 primers to create pST50Tr-STRHSTPABMBP (expresses 50.3 kD protein) to express a protein close to 50.0 kD.

7. 60 kD HSTPABPACRCC1
pPSU60 (pST50Trc2-HSTPABPACRCC1)

The internal SacI site in the RCC1 coding region in pWM529-dRCC1Δ1x3 was removed by silent PCR-based mutagenesis using STO4902/STO4903 primers to create pWM529-dRCC1Δ1x27. The BspEI-BsrGI dRCC1Δ8x27 coding region amplified from pWM529-dRCC1Δ1x27 using STO5058/STO5059 primers was subcloned into NgoMIV-BsrGI pST50Trc2-HSTPABPAC to create pST50Trc2-HSTPABPACRCC1.

8. 80 kD STRHSTPABPACIL1bRCC1
pPSU80 (pST50Tr-STRHSTPABPACIL1bRCC1)

The BamHI-NgoMIV IL1b cassette originally amplified from gBlock_IL1bS1 was subcloned into BamHI-NgoMIV pST50Tr-STRHSTPABPAC to create pST50Tr-STRHSTPABPACIL1b. The NgoMIV-BsrGI dRCC1Δ8x27 cassette originally amplified from pWM529-dRCC1Δ1x27 using STO4901/STO2393 primers was subcloned into pST50Tr-STRHSTPABPACIL1b to create pST50Tr-STRHSTPABPACIL1bRCC1.

9. 100 kD STRHSTPABPACIL1bQRS
pPSU100 (pST50Tr-STRHSTPABPACIL1bQRS)

The NgoMIV-BsrGI QRS coding region amplified from E. coli genomic DNA using STO5009/STO5010 primers was subcloned into NgoMIV-BsrGI pST50Tr-STRHSTPABGST vector to create pST50Tr-STRHSTPABGSTQRSv0. The internal MluI site in the QRS coding region was removed by silent PCR-based mutagenesis using STO5023/STO5024 primers to create pST50Tr-STRHSTPABGSTQRS. The NgoMIV-BsrGI STRHSTPABGSTQRS was subcloned into NgoMIV-BsrGI pST50Tr-STRHSTPABPACIL1b vector to create pST50Tr-STRHSTPABPACIL1bQRS.

10. 150 kD STRHSTPABMBPpepN
pPSU150 (pST50Tr-STRHSTPABMBPpepN)

The BamHI-BsrGI pepN coding region originally amplified from E. coli genomic DNA using STO4998/STO5002 primers was subcloned into BamHI-BsrGI pST50Tr-STRHSTPABMBP vector to create pST50Tr-STRHSTPABMBPpepN.

11. 250 kD STRHSTPABPACIL1bQRSSTRHSTPABMBPpepN
pPSU250 (pST50Tr-STRHSTPABPACIL1bQRSSTRHSTPABMBPpepN)

The kinased and annealed STO2260/STO2261 oligonucleotides (codes for a 12 amino acid linker) were ligated with phosphatased NgoMIV pST50Tr-STRHSTPABPACIL1bQRSv1vector to create pST50Tr-STRHSTPABPACIL1bQRSv2. The NgoMIV-BsrGI coding region amplified from pST50Tr-STRHSTPABPACIL1bQRSv2 with STO1099/STO5105 (introduces EagI and PstI sites at the QRS 3' end) was subcloned into NgoMIV-BsrGI pST50Tr-STRHSTPABPACIL1bt1QRSv2 to create pST50Tr-STRHSTPABPACIL1bt1QRS. The EagI-PstI STRHSTPABMBPpepN fragment amplified from pST50Tr-STRHSTPABMBPpepN using STO5106/STO5107 primers was subcloned into EagI-PstI pST50Tr-STRHSTPABPACIL1bt1QRS to create pST50Tr-STRHSTPABPACIL1bt1QRSSTRHSTPABMBPpepN.

12. 10-30-50-100 kD
pPSU10-30-50-100
pST44-STRHSTPABMBP-STRHSTPABPACIL1bQRS-HSTPABPACS100BCBP-STRHSTPAB

The XbaI-BglII STRHSTPABMBP insert from pST50Tr-STRHSTPABMBP was subcloned into XbaI-BglII pST44 to create pST44-STRHSTPABMBP.

The EcoRI-HindIII STRHSTPABPACIL1bQRS insert from pST50Trc2-STRHSTPABPACIL1bQRS was subcloned into EcoRI-HindIII pST44-STRHSTPABMBP vector to create pST44-STRHSTPABMBP-STRHSTPABPACIL1bQRS.

The SacI-KpnI HSTPABPACS100BCBP insert from pST50Trc3-HSTPABPACS100BCBP was subcloned into SacI-KpnI pST44-STRHSTPABMBP-STRHSTPABPACIL1bQRS to create pST44-STRHSTPABMBP-STRHSTPABPACIL1bQRS-HSTPABPACS100BCBP.

The BspEI-MluI STRHSTPAB insert from pST50Trc4-STRHSTPAB was subcloned into BspEI-MluI pST44-STRHSTPABMBP-STRHSTPABPACIL1bQRS-HSTPABPACS100BCBP vector to create pST44-STRHSTPABMBP-STRHSTPABPACIL1bQRS-HSTPABPACS100BCBP-STRHSTPAB.

13. 20-40-60-80 kD
pPSU20-40-60-80
pST44-STRHSTPABPACCBPv3-HSTPABPACGST-STRHSTPABPACIL1bRCC1-HSTPABPACRCC1

The NdeI-BsrGI STRHSTPABPACCBPv3 insert from pST50Trc4-STRHSTPABPACCBPv3 was subcloned into NdeI-BsrGI pST44 to create pST44-STRHSTPABPACCBPv3.

The EcoRI-HindIII HSTPABPACGST insert from pST50Trc2-HSTPABPACGST was subcloned into EcoRI-HindIII pST44-STRHSTPABPACCBPv3 vector to create pST44-STRHSTPABPACCBPv3-HSTPABPACGST.

The BspEI-MluI HSTPABPACRCC1 insert from pST50Trc4-HSTPABPACRCC1 was subcloned into BspEI-MluI pST44-STRHSTPABPACCBPv3-HSTPABPACGST vector to create pST44-STRHSTPABPACCBPv3-HSTPABPACGST-HSTPABPACRCC1.

The SacI-KpnI STRHSTPABPACIL1bRCC1insert from pST50Trc3-STRHSTPABPACIL1bRCC1 was subcloned into SacI-KpnI pST44-STRHSTPABPACCBPv3-HSTPABPACGST-HSTPABPACRCC1 vector to create pST44-STRHSTPABPACCBPv3-HSTPABPACGST-STRHSTPABPACIL1bRCC1-HSTPABPACRCC1.

Supplementary Note 4

Instructions for preparing the Penn State Protein Ladder.

Expression

1. Transform expression plasmid into BL21(DE3)pLysS cells. Plate onto plate media containing 100 μg/ml ampicillin and 25 μg/ml chloramphenicol. Incubate at 37°C overnight (12-18 hours) or at room temperature for 48-72 hours.

   We use the BL21(DE3)pLysS strain but other *E. coli* strains used for T7 promoter based expression should work as well.
   We use TYE plate media but LB or similar plate media should work equally well.

2. Inoculate 100 ml liquid media and 50 μg/ml ampicillin and 25 μg/ml chloramphenicol in a 500 ml Erlenmeyer flask with 3-5 colonies from a fresh transformation plate. Grow shaking at 37°C.

   We use 2xTY liquid media but LB or similar media should work as well. The culture can also be grown overnight at 21°C.

3. When OD_{600} of the culture is between 0.10 and 0.15 (typically 3 to 4 hours after inoculation), transfer to 21°C shaking incubator.

4. Induce expression by adding 0.1 ml 0.1 M IPTG when OD600 is between 0.5 and 0.8 (typically 3-4 hours after transfer to 21°C). Continue shaking at 21°C overnight (12-18 hours).

   Centrifuge 250 μl of uninduced culture in a microcentrifuge tube for 1 minute. Aspirate off supernatant and resuspend pelleted cells in 50 μl of protein gel loading buffer (PGLB). This will be the uninduced sample for SDS-PAGE.

   We prepare the gel sample from the uninduced culture used for the OD_{600} measurement.
   We anticipate inducing and growing the cells at 18 to 25°C will produce similar results to 21°C.

5. After the overnight induction at 21°C, centrifuge 100 μl of the induced culture in a microcentrifuge tube for 1 minute. Aspirate off supernatant and resuspend pelleted cells in 50 μl of PGLB. This will be the induced sample for SDS-PAGE.

   Harvest the culture by centrifuging in two 50 ml Falcon centrifuge tubes at 4000 rpm (3000 g) for 10 minutes at room temperature. Pour off the supernatant and resuspend each cell pellet in 8 ml P300 buffer.
   Store the resuspended cells at -20°C.

   You need cells from only one ~50 ml aliquot of the culture for the purification protocol below.

Purification

1. (equilibrate resin)
   For each 50 ml culture, transfer 1 ml of metal affinity resin (50% suspension) to a 15 ml Falcon centrifuge tube. Add 10 ml of deionized or MilliQ water. Mix by inversion several times. Centrifuge in tabletop centrifuge at 1800 rpm (700 g) for 2 minutes at room temperature to sediment the resin. Pour off the supernatant. Add 10 ml of P300 buffer to the resin. Mix, centrifuge and pour off supernatant as before. Cap the tube to avoid the resin drying out.
   We use ABT high density cobalt resin (Agarose Bead Technologies, 6BCL-QHCo-100) or Talon Superflow resin (Clontech, 635669) with equivalent results.

2. Thaw the frozen cell suspension by immersing tube in a beaker of water at room temperature.

3. Sonicate the thawed extract. Sample sonication conditions: Branson S-450D sonicator, 2 x 10 seconds at 40% maximum power, 50% cycle.
   Cool on ice for 10-30 seconds between sonication cycles.
   Mix 25 μl of the sonicated sample with 25 μl PGLB (for whole cell extract)

4. Centrifuge whole cell extract in 6 microcentrifuge tubes at 3 minutes at room temperature or in the cold room. Mix 25 μl of the sonication supernatant with 25 μl of PGLB (for supernatant = purification input). Transfer the supernatant to the 15 ml Falcon tube with the equilibrated metal affinity resin. Incubate for 20 minutes at room temperature on a rotator or mix by hand every 3-5 minutes.

5. Centrifuge resin-extract mixture in tabletop centrifuge at 700 g for 5 minutes at room temperature to sediment the resin. Use a pipet to transfer the supernatant (purification flow through) to a 15 ml Falcon tube labeled “flow through”. Try to transfer as much supernatant without removing resin.

6. Add 10 ml of P300 buffer to the resin. Mix by inversion several times. Centrifuge in tabletop centrifuge at 700 g for 5 min at room temperature to sediment the resin. Transfer the supernatant using a pipette or by pouring to a labeled 15 ml Falcon tube.
   Repeat this wash one more time.

7. Resuspend the washed resin in 3 ml P300 buffer. Transfer to a disposable BioSpin column (Bio-Rad, 732-6008) clamped to a retort stand and allow the liquid to drain from this mini-column. Discard this flow through.

8. Position the BioSpin column with the resin above a microcentrifuge rack containing 4 microcentrifuge tubes labeled “fr 1”, “fr 2”, “fr 3” and “fr 4”. Add 0.5 ml of P300 + 200 mM imidazole solution to the top of the resin. Collect ~0.5 ml fractions into the microcentrifuge tubes. Add additional 3 x 0.5 ml P300 + 200 mM imidazole solution in succession.

   The vast majority of the HIS tagged proteins will usually elute in fraction 1 or 2.

9. Mix 25 μl of appropriate fraction (flow through, fr 1-4) with 25 μl PGLB. Check whole cell extract, purification input, flow through and fractions on SDS-PAGE.

10. For preparing the Penn State protein ladder from individually expressed and purified proteins, we recommend combining the two most concentrated metal affinity fractions for each ladder protein and then analyzing a 1:40 dilution of this pool for each ladder protein on SDS-PAGE. The results can be used to prepare a small volume (100 – 200 μl) protein ladder before preparing the larger volume and final protein ladder.

    We prepare our protein ladder with 4x the amount of the 50 kD protein to make it easy to identify this molecular weight marker in the ladder.

<u>P300</u>	for 50 ml:
50 mM sodium phosphate pH 7.0	3.05 ml 0.5 M Na_2HPO_4<br>1.95 ml 0.5 M NaH_2PO_4
300 mM NaCl	3 ml 5 M NaCl
1 mM benzamidine	50 μl 1 M benzamidine
5 mM 2-mercaptoethanol	17 μl stock (14.4 M) 2-mercaptoethanol
	MilliQ or deionized water to 50 ml

<u>P300 + 200 mM imidazole</u>	for 10 ml:
200 mM imidazole	0.136 g imidazole<br>10 ml P300-EDTA

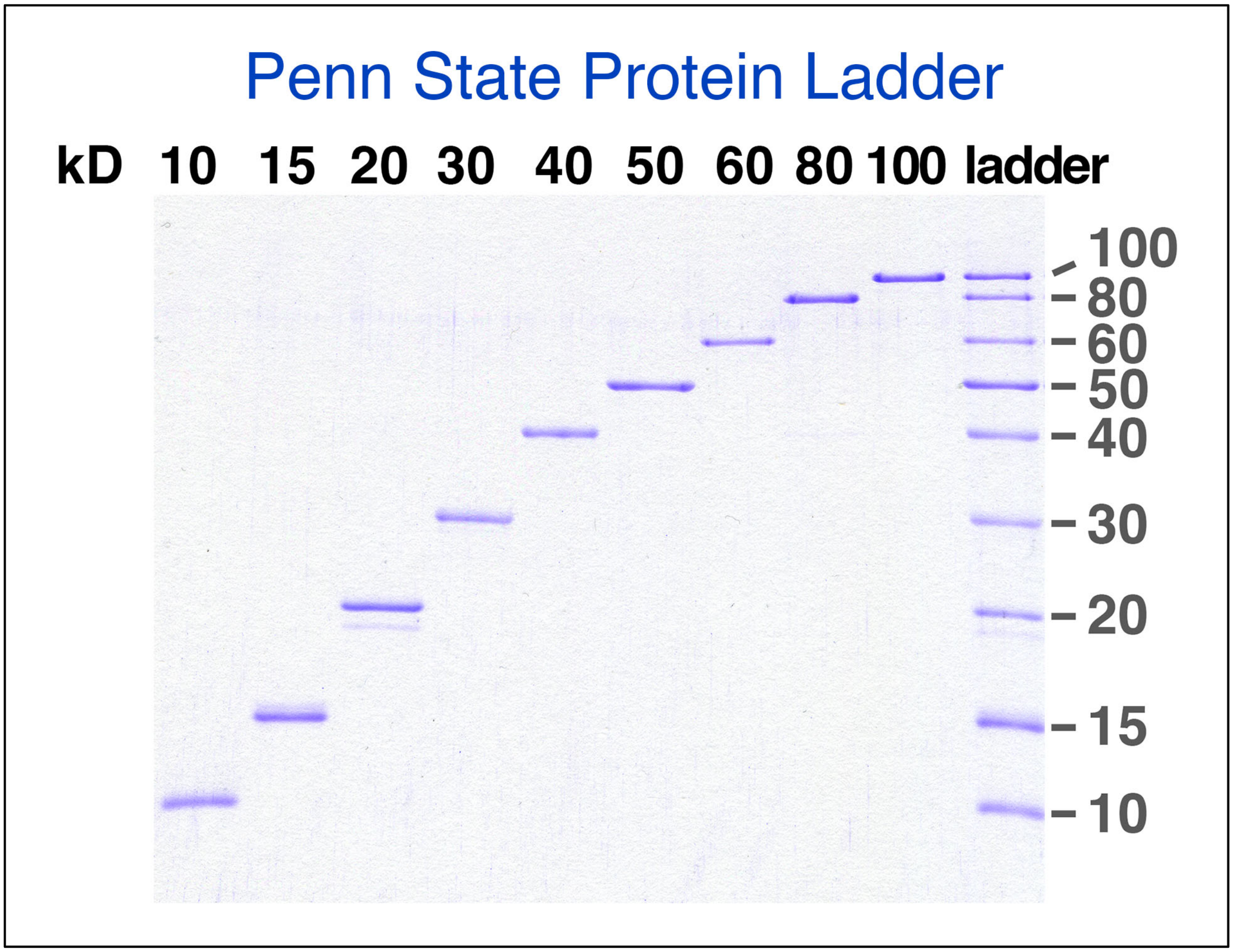

The figure above can be printed, cut along the border and inserted into a 5 inch x 7 inch photo holder
